# Optimization of Bioactive Lipid Synthesis by Enzymatic Acidolysis Using EPA + DHA Concentrate from Rainbow Trout and Tocopherols from Maqui Seed Oil

**DOI:** 10.3390/foods15030533

**Published:** 2026-02-03

**Authors:** Katherynne Mayorga, Alicia Rodríguez, Evelyn Tapia, Gretel Dovale-Rosabal, María Elsa Pando, Benjamín Claria, Melissa Tsuchida, Jenifer Sáez, Nicolás Retamal, Nalda Romero, Liliana Maier, Santiago P. Aubourg

**Affiliations:** 1 Department of Food Science and Chemical Technology, Faculty of Chemical and Pharmaceutical Sciences, University of Chile, Dr. Carlos Lorca Tobar 964, Santiago 8380494, Chilenromero@uchile.cl (N.R.); 2 Department of Nutrition, Faculty of Medicine, University of Chile, Independencia 1027, Santiago 8380000, Chile; 3 Faculty of Natural Resources and Veterinary Medicine, School of Veterinary Medicine, Santo Tomás University, Santiago 8370003, Chile; 4 Department of Food Technology, Marine Research Institute (CSIC), Eduardo Cabello 6, 36208 Vigo, Spain

**Keywords:** belly oil, *Oncorhynchus mykiss*, cold-pressed maqui seed oil, *Aristotelia chilensis* (Mol.) Stuntz, supercritical CO_2_, enzymatic acidolysis, *Candida antarctica*, optimization, EPA and DHA, tocopherols

## Abstract

A novel optimized bioactive lipid (OBL) rich in long-chain polyunsaturated omega-3 fatty acids (n-3 LCPUFA) was synthesized through enzymatic acidolysis using concentrated belly oil from rainbow trout (*Oncorhynchus mykiss*) (CB) and tocopherols obtained from cold-pressed maqui seed oil (*Aristotelia chilensis* (Mol.) Stuntz) (MSO) under supercritical CO_2_ conditions. The reaction was catalyzed by *Candida antarctica* lipase B (CALB) and optimized using a 3^2^ response surface design with 12 experimental runs and three central points, considering pressure (100–300 bar) and temperature (50–80 °C) as independent variables. The response variables included the concentrations of EPA, DHA, α-, β-, γ-, and δ-tocopherols, as well as β- and γ-tocotrienols. MSO contained 10.63, 25.62, and 53.55 g·100 g^−1^ total fatty acids (TFAs) of α-linolenic, oleic, and linoleic acids, respectively, together with 280.95 mg α-tocopherol·kg^−1^ and 89.75 mg β-tocotrienol·kg^−1^. The CB contained 49.57 g EPA + DHA·100 g^−1^ TFAs. Optimal conditions (72.7 °C and 248.9 bar), experimentally validated at the RSM-predicted point, yielded an OBL containing 41.28 g EPA + DHA·100 g^−1^ TFAs, 0.39 mg α-tocopherol·kg^−1^, 3.54 mg β-tocopherol·kg^−1^, 18.48 mg β-tocotrienol·kg^−1^, 6.92 mg γ-tocopherol·kg^−1^, and 16.36 mg γ-tocotrienol·kg^−1^. Oil quality evaluation using official AOCS methods showed that the OBL exhibited an acceptable oxidative status within international regulatory limits while retaining a measurable phenolic content and intermediate antioxidant capacity derived from MSO. This study demonstrates the successful synthesis of a stable OBL from agro-industrial by-products as a sustainable source of functional ingredients for food, nutraceutical, and cosmetic applications.

## 1. Introduction

The interest in long-chain polyunsaturated omega-3 fatty acids (n-3 LCPUFA) has increased in recent years due to their well-documented roles in promoting health and reducing the risk of chronic diseases [1]. Alpha-linolenic acid (ALA; 18:3 n-3) is the primary essential fatty acid of the n-3 family and serves as a metabolic precursor. This fatty acid can be desaturated and elongated through sequential reactions to form a series of n-3 LCPUFA [2]. ALA can be found in several plant seeds, such as flax, chia, and canola, which are considered important dietary sources of this precursor fatty acid and serve as a precursor for the synthesis of n-3 LCPUFA in the human body [1]. The main n-3 LCPUFA synthesized from ALA are eicosapentaenoic acid (EPA; 20:5 n-3) and docosahexaenoic acid (DHA; 22:6 n-3); however, it is estimated that only 8–20% of an ingested ALA dose is converted to EPA and 0.5–9% to DHA [3]. Therefore, experts from FAO-WHO/FINUT [2] recommend the direct dietary intake of n-3 LCPUFA, particularly EPA and DHA, due to their beneficial effects on cardiovascular and neurological health, respectively. These compounds are known to reduce plasma triacylglycerol (TAG) levels, improve endothelial function, and have anti-inflammatory properties; additionally, DHA plays a critical role in brain and retinal development. The main dietary sources of EPA and DHA include cold-water fish oils such as salmon, tuna, sardines, and related species [4].

Several studies have reported a progressive reduction in the availability of EPA and DHA in conventional fish oils. According to the research by Schuchardt and Hahn [5], fish oils contain approximately 18% EPA and 12% DHA. Similarly, Pando et al. [6] reported that crude and refined salmon oil contained 5.03 and 5.27 g·100 g^−1^ total fatty acids (TFAs) of EPA, and 5.32 and 5.54 g·100 g^−1^ TFAs of DHA, respectively, and when these oils were concentrated with urea, values increased to 15.49 and 14.50 g·100 g^−1^ TFAs of EPA and 17.27 and 18.26 g·100 g^−1^ TFAs of DHA. Dovale-Rosabal et al. [7] pointed out that commercial refined salmon oil contains approximately 7.53 g·100 g^−1^ TFAs of EPA and 6.25 g·100 g^−1^ TFAs of DHA. When concentrated with urea, EPA and DHA content increased to 31.20 g·100 g^−1^ TFAs and 49.31 g·100 g^−1^ TFAs, respectively. Espinosa et al. [8] indicated that refined deodorized salmon oil contained approximately 3.5 g·100 g^−1^ TFAs of EPA and 3.6 g·100 g^−1^ TFAs of DHA, and when concentrated with urea, 15.80 g·100 g^−1^ TFAs of EPA and 22.55 g·100 g^−1^ TFAs of DHA were obtained.

As a consequence of the declining EPA and DHA content in conventional fish oils, concentrates of marine oils containing up to 90% of EPA and DHA have been developed [5]. A simple, fast, and efficient technique for fatty acid concentration is complexation with urea; this option allows the handling of large amounts of raw material with relatively simple equipment; the process only requires a limited use of less toxic organic solvents, such as ethanol, which is regarded as environmentally friendly. Likewise, it is cost-effective since urea is relatively cheap; therefore, it is a simple and efficient process [9]. The methodology is based on the crystallization of urea (CO(NH_2_)_2_) into a tetragonal structure. However, in the presence of long and straight molecules, such as saturated (SFA) and monounsaturated (MUFA) fatty acids, urea forms a hexagonal lattice, whose fraction is eliminated, causing an enrichment of the non-complexing fraction, which is enriched in n-3 LCPUFA [1,7,10].

Worldwide, more than 70% of captured fish is processed for fileting, heading, or gutting. These processes generate large quantities of by-products, including heads, frames, trimmings, viscera, skin, and scales, which can represent more than 50% of the total fish biomass. By-products of some fish species, such as salmon, trout, and tuna, have been reported to be rich in EPA and DHA, making them suitable candidates for fish oil extraction and the production of n-3 fatty acid products [11]. Rainbow trout (*Oncorhynchus mykiss*), a member of the Salmonidae family, is a species native to the northwest coast of North America [12]. In salmonids, perivisceral adipose tissue and muscle are the main fat storage sites [13]. Rainbow trout belly is a by-product of the fish butchering process [9], which corresponds to the longitudinal cut obtained from the central abdominal region, including skin, bones, and connective tissues [14]. Thus, rainbow trout belly oil (RTBO) represents a renewable and sustainable source of EPA and DHA, contributing to both nutritional enhancement and environmental sustainability through the valorization of aquaculture processing residues.

N-3 LCPUFA, such as EPA and DHA, are highly susceptible to lipid oxidation due to their high degree of unsaturation. This oxidative vulnerability represents a major challenge in developing stable and bioavailable n-3 supplements and functional ingredients. Natural antioxidants such as tocopherols and carotenoids have been shown to mitigate oxidative degradation when incorporated effectively into lipid matrices. Plant extracts have been shown to delay lipid oxidation in fish and meat products during refrigerated storage, thus extending shelf life and improving quality [15,16,17].

Maqui (*Aristotelia chilensis* (Mol.) Stuntz) is a native Chilean plant species primarily recognized for its berries, which exhibit an exceptionally high polyphenol content and are considered among the most antioxidant-rich berries currently commercialized [18,19]. These berries are extensively processed by the juice industry, which utilizes approximately 70–80% of the harvested fruit and consequently generates 20–30% of solid by-products, mainly seeds [20]. Maqui seeds, which are typically separated during juice production, contain approximately 12% oil [21], indicating their potential as a secondary raw material for oil extraction. Notably, maqui seed oil contains substantial levels of tocopherols and carotenoids, which are recognized as bioactive compounds of high interest to the food, cosmetic, and pharmaceutical industries. In particular, maqui seeds exhibit elevated concentrations of α- and γ-tocopherol [22]; α-tocopherol has been associated with anti-inflammatory effects and the modulation of cholesterol metabolism [23], whereas γ-tocopherol has demonstrated strong free radical scavenging activity and potential anti-carcinogenic properties [24]. In addition to these predominant isoforms, maqui seed oil presents a diverse profile of tocopherol and tocotrienol homologs (δ-tocopherol, α-tocotrienol, β-tocotrienol, and γ-tocotrienol), whose distinct biochemical roles remain insufficiently explored when integrated as functional components in structured lipid systems.

In this context, cold-pressed maqui seed oil (MSO) was selected in the present study not only as a natural source of lipophilic antioxidants but also as a functional lipid ingredient within the structured lipid matrix. MSO naturally provides a broad spectrum of tocopherol and tocotrienol homologs with different physicochemical properties, allowing complementary antioxidant and metabolic functions to be integrated within the same lipid system. Furthermore, the combined use of MSO derived from maqui juice processing residues and oil obtained from rainbow trout belly by-products reinforces a dual waste valorization strategy, integrating agro-industrial and aquacultural residues into a circular economy approach, while delivering complementary endogenous lipophilic bioactives within the lipid matrix.

Enzymatic acidolysis enables the modification or substitution of fatty acids within the glycerol backbone, thereby improving the nutritional and physicochemical properties of triacylglycerols (TAGs). This process holds great potential for applications in the nutraceutical, pharmaceutical, food, and cosmetic industries [25]. Typically, the reaction occurs between TAGs and free fatty acids (FFAs) in the presence of a lipase catalyst [11]. In this context, enzymatic acidolysis represents an effective strategy for tailoring the fatty acid composition of TAGs, allowing the incorporation of bioactive lipids (BL) such as EPA and DHA. This approach supports the development of functional lipid matrices with added nutritional value and potential health benefits [26].

Among the available biocatalysts, *Candida antarctica* lipase B (CALB) is the most widely employed enzyme and holds the largest number of patents, due to its remarkable stability, specificity, and catalytic versatility [27]. CALB can catalyze a wide range of reactions—including hydrolysis, esterification, and alcoholysis—thereby expanding its applicability across the biofuel, food, and pharmaceutical industries [28]. Moreover, CALB exhibits a preference for non-branched long-chain fatty acids and retains high catalytic activity within a temperature range of 30–70 °C, tolerating up to 80 °C under specific conditions [29]. Additionally, its exceptional stability compared to other lipases makes it one of the most efficient biocatalysts for lipid modification processes [30]. Importantly, CALB exhibits not only regioselective capacity but also pronounced acyl-chain selectivity, enabling differential recognition and transfer kinetics of distinct bioactive lipophilic substrates based on their molecular geometry, polarity, and partition behavior in the reaction medium. This substrate selectivity is particularly relevant for understanding how CALB can simultaneously manage multiple hydrophobic substrates (EPA, DHA, and diverse tocopherol/tocotrienol isoforms) in a single synthesis step, a capability that remains largely underexplored in the literature.

Among current green technologies, supercritical fluids have attracted increasing attention due to their numerous advantages [31,32,33]. Thus, supercritical carbon dioxide (SC-CO_2_) is the most widely used solvent for enzymatic catalysis due to its similarity to organic solvents and its advantages in mass transfer and product separation. Its moderate critical conditions (7.4 MPa and 31 °C) allow the processing of thermolabile substrates such as highly oxidizable lipids [34]. Moreover, CO_2_ is generally recognized as safe (GRAS) due to its low toxicity, non-flammability, and complete removal by volatilization [35]. In enzymatic lipid modification, SC-CO_2_ can enhance substrate diffusion and product removal while enabling fine-tuning of solvent density (and therefore solubility) through controlled changes in pressure and temperature. These characteristics are particularly advantageous for systems involving multiple hydrophobic substrates, such as n-3 LCPUFA and tocopherol/tocotrienol homologs.

Despite extensive research on enzymatic transesterification for the synthesis of structured lipids, most studies have focused on relatively simple or purified lipid substrates and have predominantly relied on conventional organic solvents, even when enzyme-assisted approaches are applied [36]. While supercritical CO_2_ has emerged as a green alternative reaction medium for EPA- and DHA-enriched lipids, most reported studies primarily emphasize n-3 LCPUFA enrichment, whereas antioxidant incorporation has received comparatively less attention and is usually addressed separately. In particular, previous studies have either focused on EPA/DHA incorporation alone or on the addition of hydrophilic antioxidants [37,38], whereas lipophilic antioxidants such as tocopherols and tocotrienols have mainly been evaluated as external additives or post-synthesis supplements, rather than as integral components of the lipid architecture [39].

Consequently, a critical gap remains regarding the development and optimization of a one-step enzymatic process under SC-CO_2_ conditions capable of simultaneously incorporating EPA + DHA and a naturally diverse spectrum of tocopherol and tocotrienol homologs into the same triacylglycerol matrix using complex lipid feedstocks. The present study addresses this gap by investigating the coordinated enzymatic remodeling of lipids derived from two agro-industrial by-products: RTBO and MSO. By combining aquacultural and botanical residues within a single process, this work establishes a double waste valorization strategy and demonstrates a circular-economy approach for producing multifunctional bioactive lipids enriched in n-3 LCPUFA and endogenous lipophilic antioxidants. Importantly, this strategy directly responds to the need for sustainable lipid processing, coupling by-product valorization with a green reaction medium that minimizes organic solvent use and process complexity.

Therefore, the objective of this study was to synthesize and optimize a novel bioactive lipid (OBL) enriched in EPA + DHA and maqui-derived tocopherols and tocotrienols through CALB-catalyzed enzymatic acidolysis of a rainbow trout (*Oncorhynchus mykiss*) belly oil concentrate (CB) and cold-pressed maqui (*Aristotelia chilensis* (Mol.) Stuntz) seed oil (MSO) under SC-CO_2_ conditions, using response surface methodology (RSM). Specifically, a 3^2^ factorial design was applied to evaluate the effects of pressure and temperature on the incorporation of EPA, DHA, total EPA + DHA, and individual tocopherol and tocotrienol homologs into the resulting lipid and to determine the supercritical conditions that maximize the simultaneous transfer of these bioactive components. In parallel, the physicochemical properties of the OBL were characterized in terms of thermal behavior, lipid class distribution, color and oxidative stability, with a view to its potential application as a sustainable functional ingredient for food, nutraceutical, and cosmetic formulations.

## 2. Materials and Methods

### 2.1. Raw Materials

The study was carried out using belly tissue from rainbow trout (*Oncorhynchus mykiss*), supplied by the aquaculture company Salmones Antártica S.A. (Puerto Montt, Chile) [33]. The fish were reared and processed at the company’s facility located in Chiloé, Chile. Additionally, cold-pressed maqui seed oil (MSO) from *Aristotelia chilensis* was used, provided by De Castañas y Amores (Santiago, Chile) [33]. Both raw materials were stored at −80 °C immediately after reception until further analysis.

### 2.2. Chemical Reagents

Analytical-grade materials and chemical reagents, including glacial acetic acid (CH_3_COOH), sulfuric acid (H_2_SO_4_), celite, sodium chloride (NaCl), absolute ethanol (C_2_H_5_OH), phenolphthalein (C_20_H_14_O_4_), hexane (C_6_H_14_), potassium hydroxide (KOH), isopropanol (C_3_H_8_O), methanol (CH_3_OH), sodium methoxide (CH_3_ONa), anhydrous sodium sulfate (Na_2_SO_4_), sodium sulfite (Na_2_SO_3_), toluene (C_7_H_8_) and silica gel 60 F_254_ plates were obtained from Merck S.A. (Santiago, Chile).

High-purity gases, including zero air, liquid carbon dioxide (CO_2_) with a 99.9% purity, hydrogen (H_2_), and nitrogen (N_2_) were purchased from Gaslab-Linde (Santiago, Chile). The immobilized CALB lipase (Novozyme^®^ 435) (Bagsværd, Denmark) was supplied by Blumos S.A. (Santiago, Chile).

The standards used included gallic acid, purchased from Sigma-Aldrich (Merck S.A.), indium, methyl tricosanoate (23:0, CH_3_(CH_2_)_21_COOCH_3_) as an internal standard, the GLC-463 gas–liquid chromatography (GLC) reference standard, and triacylglycerol standards, all obtained from Nu-Chek Prep (Elysian, MN, USA). Standards from α-, β-, γ-, and δ-tocopherol and β- and γ-tocotrienol were purchased from CalbioChem^®^ (Darmstadt, Germany).

### 2.3. Extraction of Belly Oil from Rainbow Trout Using a Hexane/Isopropanol Mixture

Oil extraction followed a modified protocol previously reported [10,40,41]. Rainbow trout belly tissue was thawed at 5 °C, and 300 g of sample were chopped and ground. The ground material was homogenized with a hexane/isopropanol mixture (3:2, *v*/*v*; 1200 mL hexane and 600 mL isopropanol) for 30–60 s. The homogenate was allowed to rest for 10 min and subsequently filtered through Whatman No. 1 filter paper using a Büchner funnel under vacuum, discarding the solid residue.

A 0.47 M anhydrous Na_2_SO_4_ solution (50 g dissolved in 750 mL distilled water) was added to the aqueous filtrate, homogenized, allowed to rest for 10 min, and filtered again, discarding the Na_2_SO_4_ precipitate.

The filtrate was transferred to a separatory funnel to remove the remaining aqueous phase. Finally, the organic phase was concentrated using a rotary evaporator under reduced pressure at a temperature below 40 °C to remove the solvent and obtain the rainbow trout belly oil (RTBO).

### 2.4. Production of Free Fatty Acids (FFAs) from Rainbow Trout Belly Oil via Alkaline Hydrolysis

Free fatty acids (FFAs) were obtained following modified methods described by Dovale-Rosabal et al. [7] and Pando et al. [10]. A KOH solution was prepared by dissolving 150 g of KOH in 500 mL of distilled water and 500 mL of 95% (*v*/*v*) ethanol. This solution was mixed with 500 g of crude belly oil under reflux at 60 °C for 90 min, with constant stirring under a nitrogen atmosphere.

After cooling, 250 mL of distilled water were added, and the mixture was agitated until foam formation was observed. Unsaponifiable matter was extracted with hexane and discarded. The remaining potassium soaps were acidified to pH 1 with 6 N HCl to release FFAs, which were subsequently extracted with hexane. The hexane phase was dried over anhydrous Na_2_SO_4_, filtered through Whatman No. 1 filter paper, and concentrated under vacuum at 40 °C. Finally, the FFAs were flushed with nitrogen and stored at −80 °C until further use.

### 2.5. Urea Inclusion Complexation for the Preparation of n-3 LCPUFA Concentrate from Rainbow Trout Belly Oil

The urea inclusion complexation process was performed following the methods described by Zuta et al. [7,10,42], with minor modifications. Briefly, 35 g of fatty acids, obtained by saponification, were added to a pre-prepared solution of 252 g of urea dissolved in 95% (*v*/*v*) ethanol (932.4 mL). The mixture was subjected to continuous magnetic stirring under reflux until a homogeneous and transparent solution was obtained.

Subsequently, the solution was cooled and maintained at −22 °C with continuous stirring for 16 h to promote urea crystallization. Urea crystals were separated by vacuum filtration using Whatman No. 1 filter paper. The filtrate was acidified to pH 4.5 with 6 N HCl and washed with hexane in a separatory funnel, followed by vigorous mixing and phase separation. The upper organic phase, corresponding to the n-3 LCPUFA concentrate, was dried over anhydrous Na_2_SO_4_, filtered, and concentrated under vacuum at 40 °C using a rotary evaporator. The final concentrate was stored under a nitrogen atmosphere at −80 °C until further analysis.

### 2.6. 3^2^ Factorial Design for the Enzymatic Acidolysis of Rainbow Trout Belly Oil Concentrate and Cold-Pressed Maqui Seed Oil Under Supercritical CO_2_ Conditions

The enzymatic acidolysis reaction under supercritical CO_2_ conditions was performed following a 3^2^ full factorial design, established through response surface methodology (RSM) using Statgraphics Centurion XVI software (version 16.1.18; StatPoint Technologies, Inc., Rockville, VA, USA). Reactions were carried out in a stainless-steel reactor column (10 g total capacity) containing a mixture of 70% (*w*/*w*) n-3 LCPUFA concentrate from trout belly oil (CB), 30% (*w*/*w*) cold-pressed maqui seed oil (MSO), and 10% (*w*/*w*, relative to total lipid substrates) immobilized CALB (Novozym^®^ 435).

Enzyme loading, reaction time (2 h), and extraction time (2 h) were fixed based on previous optimization studies indicating that these conditions are sufficient to achieve near-maximum conversion and extraction efficiency in CALB-catalyzed supercritical CO_2_ systems, while minimizing mass-transfer limitations and unnecessary enzyme exposure [43].

Supercritical CO_2_ pressure (100, 200, and 300 bar) and temperature (50, 65, and 80 °C) were selected as independent variables. The response variables corresponded to the maximal incorporation levels of EPA, DHA, total EPA + DHA, tocopherols, and tocotrienols. The selected temperature range (50–80 °C) falls within the operational stability window reported for immobilized CALB in supercritical CO_2_ media, where the enzyme retains sufficient catalytic activity for lipid modification reactions despite partial thermal deactivation at the upper temperature limit [44,45].

The experimental system was assembled and operated according to the 3^2^ factorial design matrix (Table 1), comprising 12 experimental runs, including three central points to allow estimation of pure experimental error and model adequacy.

### 2.7. Optimization of Variables in the Enzymatic Acidolysis Process

Optimization of the enzymatic acidolysis process was performed by quantifying EPA and DHA contents (g·100 g^−1^ total fatty acids, TFAs) by gas–liquid chromatography (GLC) and tocopherols and tocotrienols (mg·kg^−1^ oil) by high-performance liquid chromatography (HPLC) in the bioactive lipids (BL) obtained from the 12 experimental runs.

Simultaneous multi-response optimization was carried out using RSM to identify the combination of experimental factor levels that maximized all response variables concurrently. The optimization criterion was based on the global desirability function, which ranges from 0 (undesirable) to 1 (fully desirable) [46].

Based on the experimental data, second-order polynomial regression models were developed to describe the relationship between the independent variables and the response variables, allowing determination of the theoretical optimum bioactive lipid (OBL) with maximal EPA, DHA, total EPA + DHA, and tocopherol contents. The general mathematical model (Equation (1)) derived from RSM is expressed as:

(1) $$Y=\beta_{0}+\sum_{i=1}^{k}\beta_{i}X_{i}+\sum_{i=1}^{k}\beta_{ii}X_{i}^{2}+\sum_{i=1}^{k}\sum_{j=1}^{k}\beta_{ij}X_{i}X_{j}\;+\;\varepsilon \;;i<j$$

where Y represents the response variable; β_0_, β_i_, β_ii_, and β_ij_ correspond to the intercept, linear, quadratic, and interaction coefficients, respectively; $X_{i}$ and $X_{j}$ denote the independent variables; and ε represents the random experimental error. Regression coefficients were estimated by multiple regression analysis, and only statistically significant terms (*p* < 0.05) were retained in the model. Model adequacy and the significance of regression terms were evaluated by analysis of variance (ANOVA; *p* < 0.05).

### 2.8. Physicochemical Characterization of MSO, CB, and BL

Differential scanning calorimetry (DSC) was used to evaluate the thermal behavior of MSO, CB, and OBL. Analyses were performed using a PerkinElmer 6000 MT-DSC (Waltham, MA, USA) equipped with temperature modulation. The instrument was calibrated using indium (5 mg; melting point = 156.61 °C; ΔH = 28.45 J·g^−1^) at a heating rate of 2 °C·min^−1^ under a nitrogen purge (20 mL·min^−1^). Approximately 9–10 mg of sample were hermetically sealed in aluminum volatile pans, with an empty pan used as reference. Analyses were conducted in triplicate, and results are reported as mean ± standard deviation (SD).

The thermal program consisted of cooling from 30 to −80 °C at 5 °C·min^−1^, holding for 5 min, heating to 60 °C at 5 °C·min^−1^, and maintaining for 1 min. Melting thermograms and enthalpy values (ΔH, J·g^−1^) were processed using Pyris™ Player software version 11.0.0.0449.

Lipid classes were identified by thin-layer chromatography (TLC) using silica gel 60 F_254_ plates (Merck, Santiago, Chile). The mobile phase consisted of chloroform/acetone/glacial acetic acid (96:4:1, *v*/*v*/*v*), following the method of Sabally et al. [47]. Aliquots of 1 μL were applied to the plates, and lipid classes were visualized by iodine vapor, allowing identification of monoacylglycerols (MAGs), diacylglycerols (DAGs), free fatty acids (FFAs), and triacylglycerols (TAGs).

Color parameters were evaluated using the CIELAB color space, defined by the International Commission on Illumination (CIE). The system describes color using L* (lightness), a* (green–red), and b* (blue–yellow) coordinates [48]. Color measurements were performed using a Lovibond PFX195 Series colorimeter (The Tintometer Ltd., Amesbury, UK), with samples placed in a 1.5 cm optical-path glass cuvette prior to analysis.

Fatty acid profiles of the oils were determined by GLC Shimadzu (Kyoto, Japan) after methylation according to the IUPAC [49] method to obtain fatty acid methyl esters (FAMEs). Briefly, 100 mg oil was mixed with 10 mL of 0.2 N sodium methoxide and three boiling chips, followed by heating under reflux at 95–100 °C for 10 min. After cooling, one drop of phenolphthalein and 4 mL of 4% H_2_SO_4_ in methanol were added until decolorization, followed by reheating for 30 min. The reaction mixture was cooled, extracted with 3 mL hexane and 0.5 mL methyl tricosanoate (C23:0) as internal standard, and adjusted to volume with saturated NaCl solution before storage at 4 °C.

FAMEs were analyzed using a GLC equipped with a flame ionization detector, a split injector, and an SP™-2560 capillary column (100 m × 0.25 mm × 0.2 μm; Supelco, Bellefonte, PA, USA), with hydrogen as carrier gas and 1 μL injection volume. Compound identification was performed using Data Apex Clarity™ software M021/80S (Data Apex Ltd., Prague, Czech Republic) and the GLC-463 reference standard (Nu-Chek Prep, Elysian, MN, USA) by comparing retention times. Quantification followed the official American Oil Chemistry Society (AOCS) method Ce 1j-7 [50] with modifications, and results were expressed as g fatty acid per 100 g TFAs identified.

Tocopherols and tocotrienols were identified and quantified by HPLC following the AOCS Official Method Ce 8-89 [51], using an HPLC consisting of a Merck-Hitachi pump L-6200A (Merck, Darmstadt, Germany), a Rheodyne 7725i injector with 20 μL sample loop, a LiChro-CART Superspher Si 60 column (25 cm × 4 mm id, 5 μm particle size; Merck, Darmstadt, Germany), a Hitachi Chromaster 5440 fluorescence detector, and a PC with Clarity chromatography software version 2.4.1.43 (Prague, Czech Republic). The mobile phase consisted of propan-2-ol in hexane (0.5:99.5, *v*/*v*) at a flow rate of 1 mL·min^−1^. Calibration was performed using Calbiochem (Santiago, Chile) standards of α-, β-, γ-, and δ-tocopherols and β- and γ-tocotrienols. An injection volume of 80 μL was used, and detection was carried out at 290 and 330 nm. Tocopherol identification was based on comparing the retention times of sample peaks with those of reference standards. Quantification was performed according to the relationship (Equation (2)):

(2) $$Tocopherols\;and\;tocotrienols\;concentration\;(mg\cdot {kg}^{-1}oil)=\frac{C\;\times \;a\;\times \;v\;}{A\;\times \;m}$$

where *C* is the standard concentration (μg·mL^−1^), *a* is the sample peak area (mV·s), *V* is the flask volume (mL), *A* is the standard peak area (mV·s), and *m* is the sample mass (kg).

Oil quality parameters were evaluated using official AOCS methods [52], including free fatty acid content (Ca 5a-40), peroxide value (Cd 8b-90), *p*-anisidine value (Cd 18-90), and total oxidation value (TOTOX; Cg 3-91). Lipid oxidation damage was further assessed by measuring conjugated diene and triene presence at 233 and 268 nm, respectively (Ti 1a-64).

Total phenolic content was determined using the Folin–Ciocalteu colorimetric method, as described by Fuentes et al. [53]. Oil samples (0.5 g) were extracted with a methanol–water mixture (80:20, *v*/*v*) assisted by vortexing and sonication, followed by centrifugation. An aliquot of the extract was reacted with Folin–Ciocalteu reagent and sodium carbonate, and absorbance was measured at 725 nm using a UV–Vis spectrophotometer. Quantification was performed using a gallic acid calibration curve, and results were expressed as µg gallic acid equivalents per gram of oil (µg GAE·g^−1^). All analyses were conducted in triplicate.

The antioxidant capacity was determined using the hydrophilic oxygen radical absorbance capacity (H-ORACFL) assay as described by Fuentes et al. [53]. Oil samples were extracted with a methanol–water mixture (80:20, *v*/*v*), and the resulting extracts were analyzed using fluorescein as the fluorescent probe in 0.075 M phosphate buffer (pH 7.0). Trolox calibration solutions (12.5–100 µM) were used as standards. The assay was performed in black 96-well microplates by adding 25 µL of sample extract or Trolox standard and 150 µL of fluorescein solution. After incubation at 37 °C for 30 min, the reaction was initiated with 25 µL of AAPH solution (150 mM). Fluorescence was recorded at 485 nm (excitation) and 528 nm (emission). Antioxidant capacity was calculated from the area under the fluorescence decay curve and expressed as µmol Trolox equivalents per gram of oil (µmol TE·g^−1^). All analyses were conducted in triplicate.

### 2.9. Statistical Analysis

Results are expressed as mean ± standard deviation (SD), and analysis of variance (ANOVA) was performed. Statistical analyses were conducted using Statgraphics Centurion XVI-2011 software 16.1.18 (StatPoint Technologies, Inc., Rockville, VA, USA).

## 3. Results

### 3.1. Effect of Independent Variables of Enzymatic Acidolysis Process on Response Variables

The enzymatic acidolysis in a supercritical CO_2_ medium was carried out using a 3^2^ experimental design based on the RSM, which allowed the evaluation of different combinations of supercritical CO_2_ pressure (bar) and temperature (°C) in order to optimize the synthesis of a BL. A total of twelve experiments were performed. The independent variables considered were supercritical pressure and temperature, while the response variables corresponded to the contents of EPA, DHA, and EPA + DHA, as well as the contents of tocopherols and tocotrienols.

Table 2 presents the effects of supercritical temperature and pressure on the EPA, DHA, and EPA + DHA contents, whereas Table 3 shows the effects of such independent variables on the α-tocopherol, β-tocopherol, β-tocotrienol, γ-tocopherol, γ-tocotrienol, and δ-tocopherol contents.

The ANOVA for each response variable is presented in Table 4, using the mean square residual method, which allows the determination of the statistical significance of the independent variables and their interactions. For responses showing a significant lack of fit, the model is used only to identify trends and approximate optima.

The variables that showed statistically significant effects were DHA, EPA + DHA, α-tocopherol, β-tocopherol, and γ-tocotrienol values. The significant effects (*p* < 0.05) were distributed as follows: pressure (A) influenced the levels of DHA and β-tocopherol, while temperature (B) affected the contents of DHA and α-tocopherol. The quadratic term of pressure (A^2^) was significant for the concentrations of EPA + DHA and α-tocopherol. In contrast, the quadratic term of temperature (B^2^) showed statistical significance for all variables except for β-tocopherol.

**Figure 1:**
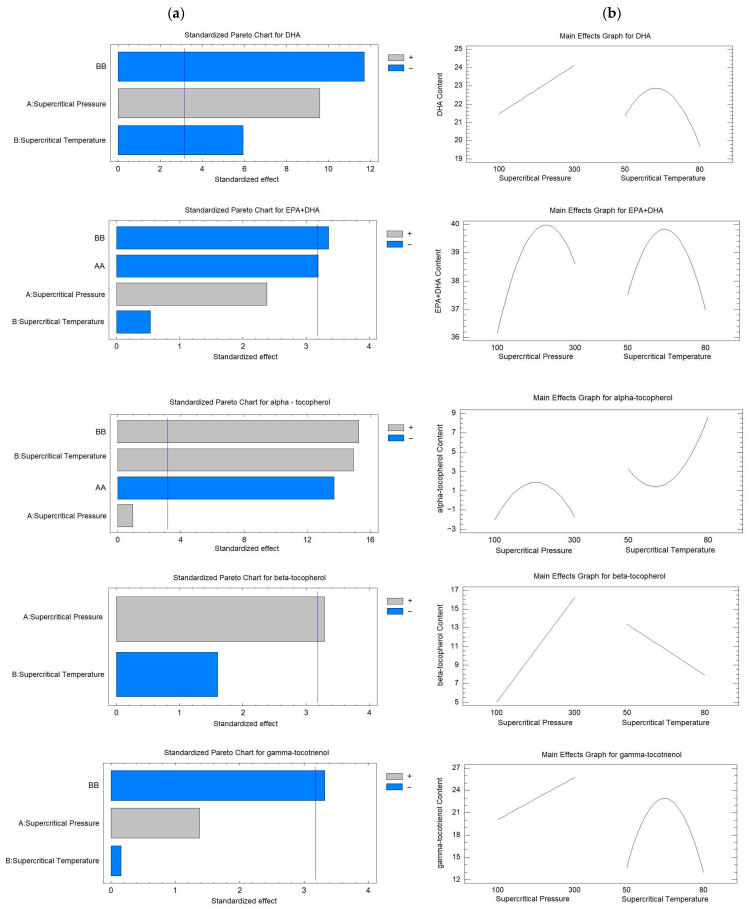
(**a**) Standardized Pareto charts and (**b**) graphs of the main effects affecting the concentration of the response variables. Figure 1a shows the independent variables and their combinations that significantly affect the concentration of the response variables (*p* < 0.05). Gray bars represent factors with a positive effect on the response, while blue bars indicate variables and interactions with a negative effect. The blue vertical line placed to the right denotes the threshold for statistical significance (*p* < 0.05). Figure 1b represents the behavior of the main effects on the response variables.

To analyze the effect of supercritical CO_2_ pressure and temperature, standardized Pareto charts and main effect plots were constructed for the statistically significant response variables. Figure 1a presents the Pareto charts, which highlight the main independent variables influencing the enzymatic acidolysis process for the synthesis of the BL. Linear (A, B) and quadratic (A^2^, B^2^) effects were evaluated for each response variable using a statistical significance threshold of *p* < 0.05. In addition, the main effects for DHA, EPA + DHA, α-tocopherol, β-tocopherol, and γ-tocotrienol (Figure 1b) were analyzed to interpret the individual influence of each factor.

According to Figure 1a, increasing pressure (A) exerted a significant and positive effect on DHA and β-tocopherol concentrations. This indicates that higher pressure during enzymatic acidolysis enhances the incorporation of these compounds into the BL (Figure 1b).

Regarding temperature (B), a significant negative effect was observed for DHA, as illustrated in Figure 1b. This effect followed an inverse parabolic trend, where DHA concentration initially increased up to a maximum point and subsequently decreased as temperature continued to rise. Conversely, temperature showed a significant positive effect on α-tocopherol, exhibiting a parabolic behavior characterized by an initial decrease to a minimum point, followed by an increase with further temperature elevation (Figure 1b).

Moreover, the quadratic term of pressure (A^2^) displayed a significant negative effect on EPA + DHA and α-tocopherol concentrations. As shown in Figure 1b, this response followed an inverted parabolic pattern, indicating that beyond the optimal pressure, the concentrations of these compounds decreased as the influence of the quadratic pressure became more pronounced.

**Figure 2:**
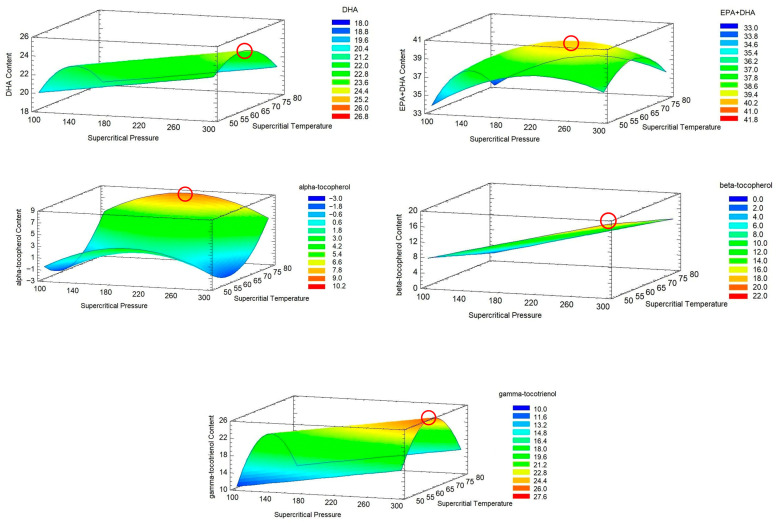
Estimated response surface graphs of the combination of independent variables supercritical CO_2_ pressure and temperature on the response variables DHA, EPA + DHA, α-tocopherol, β-tocopherol, and γ-tocotrienol. The red circle indicates the optimal region of the response surface.

Three-dimensional response surface plots indicate the combined effects of supercritical pressure and temperature on DHA, EPA + DHA, α-tocopherol, β-tocopherol, and γ-tocotrienol values (Figure 2). These plots reveal that the interaction between both variables significantly influences the response profiles, from yellow to red regions, this indicating the maximum predicted concentrations.

From the RSM analysis, mathematical equations were obtained to describe the behavior of each compound as a function of the independent variables pressure (A) and temperature (B). These models quantitatively predict the responses within the experimental range and serve as the basis for multivariable optimization. The resulting equations (Equations (3)–(7)) include linear terms (A and B), as well as quadratic components such as the quadratic term of pressure (A^2^) and the quadratic term of temperature (B^2^), indicating the presence of nonlinear effects in the system. The estimated coefficients reflect the direction and magnitude of the influence of each variable, while the quadratic terms denote curvature effects associated with the processing conditions. The models achieved coefficients of determination (R^2^) of 47.77%, 40.68%, 51.42%, 45.70%, and 36.37% for DHA, EPA + DHA, α-tocopherol, β-tocopherol, and γ-tocotrienol, respectively, indicating a moderate fit consistent with the complexity of the lipid matrix. Although the R^2^ values obtained for several response surface models ranged from moderate to low (≈36–52% for certain compounds), this behavior is consistent with the inherent complexity of modeling multi-component lipid systems under supercritical conditions. The simultaneous incorporation of EPA, DHA and multiple tocopherol/tocotrienol homologs involves competing phenomena, including differential substrate solubilization, mass transfer limitations, isoform-selective enzymatic kinetics, and potential oxidative or thermal degradation pathways, all of which contribute to increased model residual variance. Consequently, these empirical models should be interpreted primarily as tools to identify significant trends, locate approximate optima, and guide process development rather than as precise quantitative predictors. Despite the moderate goodness-of-fit, the models successfully identified statistically significant effects (pressure, temperature, and quadratic terms) and provided practical optimization guidance, as validated by the acceptable prediction errors observed at the predicted optimum.

DHA = −19.1726 + 0.0132333 × A + 1.26459 × B − 0.0101481 × B^2^ (3)

EPA + DHA = −19.7592 + 0.110367 × A + 1.4745 × B − 0.000245375 × A^2^ − 0.0114833 × B^2^ (4)

α-tocopherol = 52.3894 + 0.149412 × A − 2.19884 × B − 0.000369137 × A^2^ + 0.0182894 × B^2^ (5)

β-tocopherol = 11.2579 + 0.056225 × A − 0.182533 × B(6)

γ-tocotrienol = −162.579 + 0.0284167 × A + 5.55496 × B − 0.0428963 × B^2^ (7)

### 3.2. Optimization of the Variables of the Enzymatic Acidolysis Process for CB and MSO Using Supercritical CO_2_ to Obtain a BL

Table 5 (Part a) summarizes the optimization of the enzymatic acidolysis process variables, including supercritical CO_2_ temperature (°C) and pressure (bar), based on the response variables data for DHA, EPA + DHA, α-tocopherol, β-tocopherol, and γ-tocotrienol.

Model validation at the predicted optimum (248.9 bar, 72.7 °C) showed acceptable agreement between experimental and predicted values for the main response variables. EPA + DHA incorporation reached 41.28 g·100 g^−1^ TFAs, compared to a predicted value of 39.01 g·100 g^−1^ TFAs, corresponding to a prediction error of 5.8%, confirming the practical reliability of the model for global n-3 LCPUFA enrichment. DHA content exhibited a moderate prediction error of 12.9% (25.31 vs. 22.42 g·100 g^−1^ TFAs). In contrast, substantially higher prediction errors were observed for individual tocopherol and tocotrienol homologs, including 24.2% for γ-tocotrienol (16.36 vs. 21.58 mg·kg^−1^ oil), 70.5% for β-tocopherol (3.54 vs. 11.98 mg·kg^−1^ oil), and 89.0% for α-tocopherol (0.39 vs. 3.53 mg·kg^−1^ oil). These discrepancies reflect the intrinsic variability of complex multi-component lipid systems under supercritical CO_2_ conditions, where differential substrate solubility, competitive enzymatic kinetics, and potential thermal or oxidative degradation contribute to increased model uncertainty. Overall, these results support the use of the RSM models primarily as tools to identify significant trends and approximate optimal regions rather than as precise quantitative predictors for all individual responses.

### 3.3. Multiple Response Optimization of Enzymatic Acidolysis and Desirability

Table 5 (Part b) presents the optimal combination of factor levels corresponding to 248.9 bar and 72.7 °C. Under these conditions, the predicted concentrations were 22.42 g·100 g^−1^ TFA for DHA, 39.01 g·100 g^−1^ TFAs for EPA + DHA, 3.53 mg·kg^−1^ oil for α-tocopherol, 11.98 mg·kg^−1^ oil for β-tocopherol, and 21.58 mg·kg oil for γ-tocotrienol.

**Figure 3:**
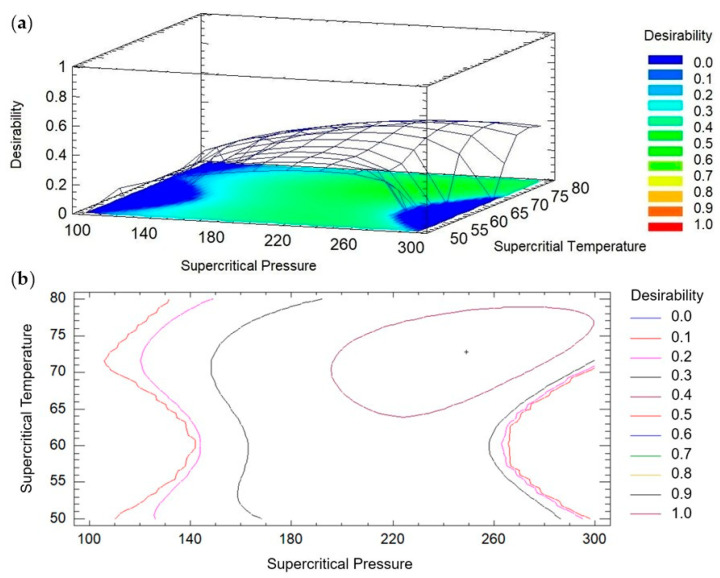
Multivariate optimization of bioactive compounds under supercritical CO_2_ conditions. (**a**) Response surface plots illustrating the joint optimization of DHA, EPA + DHA, α-tocopherol, β-tocopherol, and γ-tocotrienol. (**b**) Contour plot showing the optimal combination of supercritical CO_2_ temperature and pressure that maximizes the overall desirability function of Figure 3a. The point indicates the optimal region corresponding to 248.9 bar and 72.7 °C.

Since each compound exhibited distinct behavior under different supercritical CO_2_ pressure and temperature conditions, the Derringer desirability function was applied to achieve multi-response optimization. As shown in Figure 3, the system exhibits high sensitivity, since slight increases or decreases in pressure and temperature near the design limits result in pronounced changes in the concentration of the target compounds. In this experimental design, the multiple response optimization showed an overall desirability value of 0.44, as determined by the STATGRAPHICS Centurion XVI Version 16.1.03 (32-bit) (Leesburg, VA, USA). The obtained desirability value indicates a moderate fit of the model to the established optimization criteria, suggesting that the selected conditions allow the simultaneous maximization of the responses within the evaluated experimental range.

### 3.4. Experimental Validation of the OBL Formulation

Table 5 (Part c) presents the experimental validation of the multiple response optimization corresponding to the response variables shown in Part b. BL was validated using the optimized independent variables, namely 248.9 bar (supercritical CO_2_ pressure) and 72.7 °C (supercritical CO_2_ temperature). Under these conditions, the following experimental results were obtained: 25.31 g·100 g^−1^ TFAs for DHA, 41.28 g·100 g^−1^ TFAs for EPA + DHA, 0.39 mg·kg^−1^ oil for α-tocopherol, 3.54 mg·kg^−1^ oil for β-tocopherol, and 16.36 mg·kg^−1^ oil for δ-tocopherol.

### 3.5. Characterization of Physicochemical Properties of CB, MSO, and OBL

#### 3.5.1. Differential Scanning Calorimetry (DSC)

**Figure 4:**
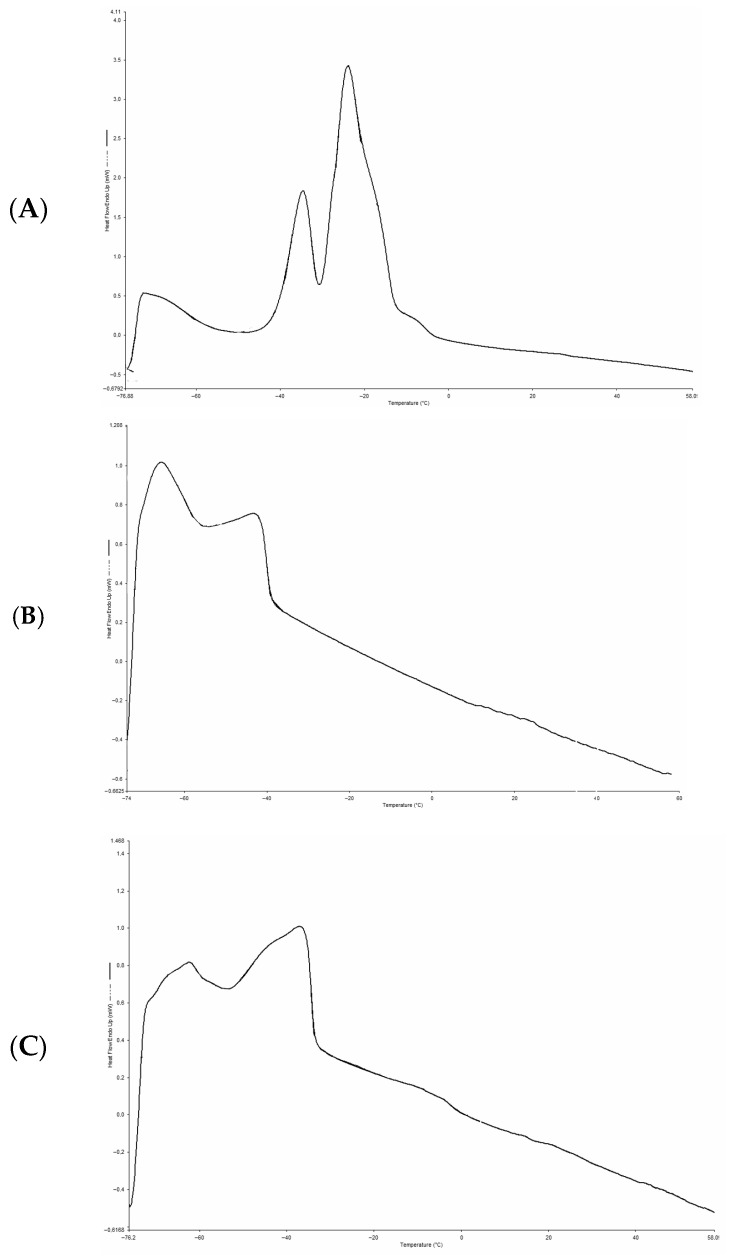
(**A**) Cold-pressed maqui seed oil (MSO) fusion thermogram (**B**) n-3 LCPUFA concentrates from trout belly oil (CB) fusion thermogram (**C**) Optimal bioactive lipid (OBL) fusion thermogram.

The DSC thermograms of the melting profiles are shown in Figure 4. The melting range of MSO was −75.59 to −15.56 °C, with an enthalpy of 37.45 J·g^−1^, whereas the CB exhibited a melting range of −71.09 to −39.24 °C and an enthalpy of 5.73 J·g^−1^. Three endothermic peaks were observed for the MSO (Figure 4A) and two for the CB (Figure 4B). The optimized BL (Figure 4C) showed a melting range between −73.53 and −1.24 °C with an enthalpy of 10.42 J·g^−1^. Three thermal peaks were detected, indicating the presence of different triacylglycerol fractions with varying degrees of saturation and fatty acid chain lengths.

Table 6 compares the thermal behavior of MSO, CB, and OBL. The calorimetric parameters of the optimized lipid showed intermediate values between those of the raw materials. In particular, the melting enthalpy (10.42 J·g^−1^) was found intermediate between that one of maqui seed oil (37.45 J·g^−1^) and that of the n-3 LCPUFA concentrate (5.73 J·g^−1^); this result suggests a redistribution of TAGs as a result of the enzymatic acidolysis process.

**Figure 5:**
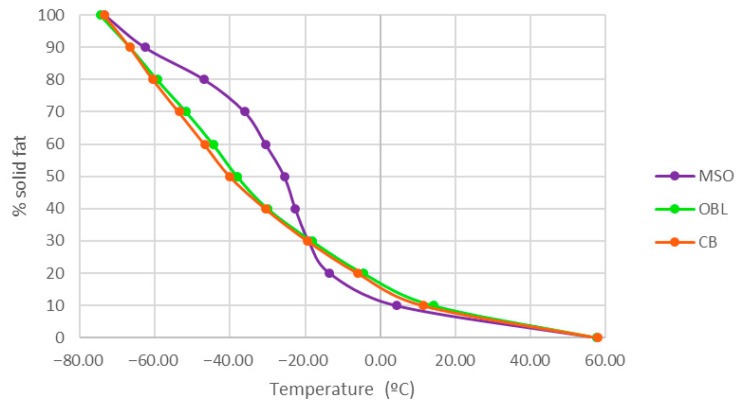
Comparison of the variation in the percentage of solid fat as a function of temperature for cold-pressed maqui seed oil (MSO), n-3 LCPUFA concentrate from trout belly oil (CB) and optimal bioactive lipid (OBL).

Figure 5 shows the variation in the solid fat content (SFC) as a function of the temperature for the three lipid samples. MSO exhibited a higher solid fat content at low temperatures, indicating a greater proportion of high-melting compounds. In contrast, CB displayed an earlier melting behavior, while OBL showed an intermediate profile between both oils.

#### 3.5.2. Thin-Layer Chromatography (TLC) Analysis of Lipid Class Distribution

**Figure 6:**
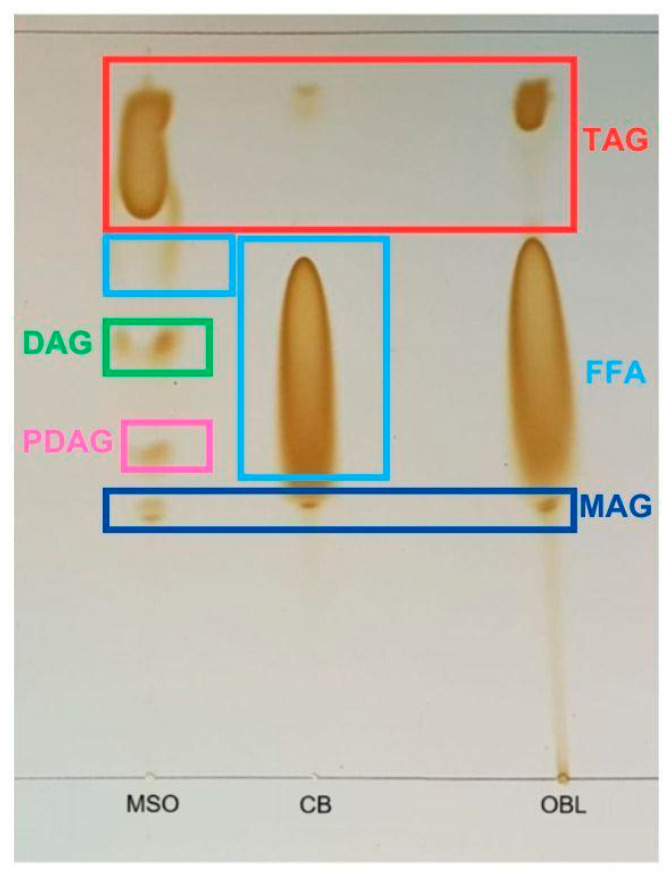
Thin-layer chromatography (TLC) of cold-pressed maqui seed oil (MSO), n-3 LCPUFA concentrate from trout belly oil (CB) and optimal bioactive lipid (OBL). The figure shows the separation of monoacylglycerols (MAGs), diacylglycerols (DAGs), phenolic diacylglycerols (PDAGs), free fatty acids (FFAs), and triacylglycerols (TAGs). Mobile Phase: chloroform/acetone/acetic acid (96:4:1, *v*/*v*/*v*).

Figure 6 shows the characterization of lipid species by TLC, employed to verify the synthesis of acylglycerols. Bands corresponding to MAGs, DAGs, phenolic diacylglycerols (PDAGs), TAGs, and FFAs were observed, along with the respective standards. The separation of these compounds occurred according to their polarity, in agreement with the findings reported by Dovale-Rosabal et al. [45], allowing clear differentiation of the lipid species present in the analyzed samples.

#### 3.5.3. Measurement of Color Parameters (L*, a*, and b*)

The results obtained from the measurement of the color parameters L*, a*, and b* are shown in Table 7. Significant differences (*p* < 0.05) were observed between MSO, CB, and OBL for the values L*, a* and b*.

The L* parameter represents lightness, ranging from white to black along the vertical (Y) axis. The a* value indicates the chromatic transition from green to red on the X-axis, while b* describes the color variation from yellow to blue on the Z-axis [40].

#### 3.5.4. Analysis of the Fatty Acid Profile

The fatty acid composition results obtained by GLC for the MSO, CB and the OBL are presented in Table 8, expressed as g fatty acids·100 g^−1^ TFAs, according to the AOCS Ce 1j-07 procedure [42].

In the MSO, the predominant fatty acids were linoleic acid (53.55 ± 0.74 g·100 g^−1^ TFAs), oleic acid (25.62 ± 0.12 g·100 g^−1^ TFAs) and α-linolenic acid (10.63 ± 0.18 g·100 g^−1^ TFAs).

In the CB, the main fatty acid were EPA + DHA (49.57 g·100 g^−1^ TFAs), DHA (30.56 ± 0.02 g·100 g^−1^ TFAs), linoleic acid (19.48 ± 0.16 g·100 g^−1^ TFAs) and EPA (19.01 ± 0.03 g·100 g^−1^ TFAs). In the OBL, the predominant fatty acids were EPA + DHA (41.28 g·100 g^−1^ TFAs), DHA (25.31 ± 0.38 g·100 g^−1^ TFAs) and EPA (15.97 ± 0.27 g·100 g^−1^ TFAs).

#### 3.5.5. Determination of Tocopherol and Tocotrienol Compounds

The identification and quantification of tocopherols and tocotrienols were performed by high-performance liquid chromatography with ultraviolet detection (HPLC-UV) and expressed as mg·kg^−1^ oil. The compounds detected included α-tocopherol, β-tocopherol, γ-tocopherol, γ-tocotrienol, and β-tocotrienol, as shown in Table 9.

In the MSO, the predominant tocochromanols, in decreasing order of concentration, were α-tocopherol (280.95 mg·kg^−1^), β-tocotrienol (89.75 mg·kg^−1^), δ-tocopherol (10.60 mg·kg^−1^), and β-tocopherol (9.17 mg·kg^−1^). The CB exhibited γ-tocopherol (7.40 mg·kg^−1^), γ-tocotrienol (20.79 mg·kg^−1^), δ-tocopherol (22.00 mg·kg^−1^), and α-tocopherol (103.85 mg·kg^−1^). In the OBL, α-tocopherol (0.39 mg·kg^−1^), β-tocopherol (3.54 mg·kg^−1^), γ-tocopherol (6.92 mg·kg^−1^), γ-tocotrienol (16.36 mg·kg^−1^), and β-tocotrienol (18.48 mg·kg^−1^) were identified and quantified (Table 9).

#### 3.5.6. Oxidative Quality Parameters of the Analyzed Lipid Samples

The quality analysis results of the optimal bioactive lipid are presented in Table 10. The peroxide value was 5.61 mEq O_2_·kg^−1^ oil, which is below the maximum limit established by the Chilean Food Sanitary Regulation (i.e., <10 mEq O_2_·kg^−1^ oil) and slightly above the limit defined by the Codex Alimentarius for fish oils (i.e., ≤5 mEq O_2_·kg^−1^ oil).

Conjugated diene and triene values were 0.0063 and 0.0047, respectively. The *p*-anisidine value was 10.08, remaining below the maximum value established by the Codex Alimentarius Commission for fish oils (i.e., ≤20). The total oxidation (TOTOX) value reached 21.3, which is also within the limit established by the Codex Alimentarius Commission for fish oils (i.e., ≤26). Compared to the individual oils used as raw materials, the optimal bioactive lipid exhibited higher TOTOX values.

#### 3.5.7. Total Phenolic Content of Oil Samples

The total phenolic content calculated using the gallic acid standard curve is presented in Table 11. The OBL showed a total phenolic content of 0.743 ± 0.22 mg gallic acid equivalents (GAE)·g^−1^ oil, while maqui seed oil exhibited a value of 2.871 ± 0.01 mg GAE·g^−1^ oil. In contrast, the CB presented a markedly lower total phenolic content (0.028 ± 0.43 mg GAE·g^−1^ oil).

#### 3.5.8. Antioxidant Capacity of Oil Samples Determined by H-ORAC Assay

As shown in Table 11, MSO presented an antioxidant capacity of 4.85 ± 0.00 µmol Trolox equivalents (TE)·g^−1^ oil, whereas the OBL exhibited a lower H-ORAC value of 1.08 ± 0.00 µmol TE·g^−1^ oil (Table 12). The CB was not determined under the conditions of the assay.

## 4. Discussion

### 4.1. Optimization of Enzymatic Acidolysis Conditions Under Supercritical CO_2_

In this study, a BL enriched with EPA, DHA, and tocopherols was synthesized from CB and MSO using CALB-catalyzed acidolysis under supercritical CO_2_ conditions. The process, optimized using a 3^2^ response surface design, revealed that both pressure and temperature significantly affected the incorporation of DHA, EPA + DHA, α-tocopherol, β-tocopherol, and γ-tocotrienol in the BL. While the model predicted a DHA content of 22.42 g·100 g^−1^ TFAs and an EPA + DHA content of 39.01 g·100 g^−1^ TFAs, the higher experimental values of DHA and EPA + DHA in the validation process indicated high catalytic efficiency of the enzyme system (Table 5c). This result is consistent with the substrate selectivity of CALB, which prefers unsaturated to saturated fatty acids [54], and with the reported thermal stability between 60 and 80 °C [55]. In contrast, tocopherol and tocotrienol concentrations were lower than expected, likely due to the nonlinear response of the system and the combined effects of high supercritical CO_2_ pressure and temperature that may influence the selectivity of the lipase.

Although the temperature range employed (50–80 °C) falls within the reported stability limits of immobilized CALB in supercritical CO_2_ systems, partial enzyme deactivation cannot be excluded, particularly near 80 °C where both thermal stress and potential substrate oxidation may contribute to reduced catalytic efficiency. The observed parabolic response profiles for several compounds (e.g., DHA and α-tocopherol) may partly reflect this interplay between enhanced kinetics at intermediate temperatures and cumulative losses in enzyme activity and substrate integrity at higher temperatures. Future studies should quantify CALB reusability and long-term stability through repeated reaction cycles under optimized supercritical conditions to fully assess process robustness and economic viability.

The consistently positive effect of pressure on DHA and β-tocopherol incorporation can be mechanistically explained by the increase in supercritical CO_2_ density and solvating power with rising pressure. For hydrophobic solutes, solubility in SC-CO_2_ generally increases at constant temperature as pressure increases due to the associated rise in CO_2_ density and solvent strength [56,57,58,59]. This behavior has been reported for a wide range of lipophilic compounds, including pharmaceuticals, oils, and lipid fractions, supporting its relevance to complex lipid systems [56,57,58,59]. As CO_2_ density increases, the supercritical phase more effectively penetrates viscous lipid matrices, reduces interfacial tension and apparent viscosity, and enhances mass transfer toward immobilized enzyme particles [56,59]. These effects collectively improve substrate availability and accessibility to CALB active sites, thereby favoring the incorporation of highly hydrophobic substrates such as DHA and β-tocopherol under higher-pressure conditions.

The parabolic temperature dependence observed for DHA incorporation in CALB-catalyzed systems reflects a critical balance between enhanced enzymatic activity and increasing oxidative and thermal instability. Within the intermediate temperature range (~60–70 °C), higher temperatures promote CALB catalytic efficiency by improving molecular mobility, mass transfer, and enzyme–substrate interactions in biphasic and supercritical media [60]. However, beyond this optimal window (~80 °C), these benefits are progressively counteracted by partial thermal deactivation of immobilized CALB, reduced active-site flexibility, and loss of catalytic efficiency [60]. Simultaneously, the highly polyunsaturated structure of DHA renders it particularly vulnerable to temperature-induced oxidation and degradation, which becomes more pronounced at elevated temperatures [61]. As a result, maximum DHA incorporation is achieved at intermediate temperatures where enzymatic performance remains high while oxidative degradation of DHA is still limited. In contrast, chemically more stable lipid components such as β-tocopherol exhibit less marked temperature dependence, consistent with their lower susceptibility to thermal oxidation [61].

### 4.2. Comparison of the Characterization of Physicochemical Properties of CB, MSO, and OBL

DSC is a widely used technique to evaluate the thermal behavior and physicochemical properties of lipid systems. In this study, the MSO exhibited a thermal profile consistent with that reported by Reinoso et al. [33], showing an onset temperature of −77.47 °C and three endothermic peaks at −73.70 °C, −33.74 °C, and −22.72 °C. This behavior aligns with observations in highly unsaturated vegetable oils, where lower melting peaks (around −38 °C) correspond to highly unsaturated TAGs, whereas higher temperature peaks (around −25 to −13 °C) are associated with molecules with a lower degree of unsaturation [62].

The CB exhibited a lower number of thermal peaks, which can be attributed to the removal of SFAs during the urea complexation process, as these compounds are responsible for melting at higher temperatures [63]. The peaks detected correspond mainly to n-3 LCPUFAs, as confirmed by GLC analysis. In the thermogram, the first melting peak was observed at −66.26 ± 0.48 °C, suggesting the presence of EPA and DHA. Similar results were reported by Berríos et al. [9] for concentrated salmon oil, showing a melting range between −75.34 and −41.81 °C, which is comparable to the range obtained in this study (−72.09 to −42.28 °C). According to Turchini et al. [64], both chain length and degree of unsaturation are critical determinants of fatty acid melting points, which increase with longer chain lengths and lower unsaturation levels, this fact explaining the behavior observed.

The melting thermogram of the OBL obtained by DSC exhibited a melting range between −73.53 and −1.24 °C, with an enthalpy of 10.42 J·g^−1^. Three thermal peaks were identified, indicating the presence of different TAG fractions with varying degrees of saturation and chain length. TAGs, the main components of the lipid system, consist of a glycerol backbone esterified with three fatty acids, whose chain length, degree of saturation, and positional distribution determine the stereochemical structure of the molecule. Moreover, molecular interactions such as glycerol conformation, aliphatic chain packing, and methyl-end stacking significantly influence the polymorphic structures of TAGs and, consequently, their thermal behavior [65]. In this context, the heterogeneity observed in the thermogram suggests the coexistence of multiple TAG species formed during the enzymatic acidolysis reaction. The three detected peaks were less defined than those observed in the thermograms of MSO and CB, indicating a redistribution of fatty acids within the TAG matrix as a result of the acidolysis process. From an application perspective, the modified melting profile and reduced enthalpy (ΔH) of the OBL relative to the starting CB and MSO suggest improved suitability for functional food and nutraceutical formulations. The broader melting range and lower crystallization temperature of OBL facilitate easier handling and processing at refrigeration temperatures (4–10 °C) compared to more solid-like fish oil concentrates, while the intermediate melting point supports controlled release behavior in gastrointestinal environments or encapsulation matrices. These thermal characteristics suggest potential suitability of OBL for applications such as microencapsulation in dairy products, soft gels, or lipid-based delivery systems, where phase transitions must align with processing and physiological conditions without compromising stability or sensory attributes [66,67,68,69,70,71]. However, further formulation and stability studies are required to validate these applications.

The variation in SFC as a function of temperature showed marked differences among the lipid samples. MSO exhibited a higher SFC at low temperatures, attributable to its high proportion of linoleic acid and lower content of n-3 LCPUFA (EPA and DHA), which favor the formation of TAGs with higher melting points and a more stable crystalline network. In contrast, the CB displayed a lower SFC and an earlier onset of melting, consistent with its elevated EPA and DHA levels, which reduce the crystallization tendency and enhance the oil fluidity. Lipids rich in unsaturated fatty acids generally exhibit lower melting points and slower nucleation rates [65]. OBL, obtained through enzymatic modification, presented an intermediate behavior between both oils; this fact reflects a partial redistribution of fatty acids within the TAG matrix, which conferred intermediate thermal properties [72].

Regarding the TLC analysis, in MSO the first compounds to migrate were TAGs, followed by DAGs, PDAGs, and MAGs, consistent with previous reports [73]. In CB, a predominant band of FFAs was observed, accompanied by minor amounts of TAGs and MAGs, confirming the efficiency of the concentration process. The relative band intensities observed on TLC plates provide semi-quantitative evidence of enzymatic acidolysis, as reflected by changes in lipid class distribution compared to the starting materials. Specifically, OBL exhibited a dominant TAG band with moderate DAG content and reduced MAG/FFA signals relative to the substrate mixture, indicating effective acyl exchange and re-esterification during the enzymatic process. TLC therefore serves as complementary visual evidence supporting lipid remodeling under the optimized supercritical CO_2_ conditions, in agreement with the compositional changes detected by GLC and HPLC analyses.

The lipid profile of OBL, as evaluated by TLC, revealed the predominance of TAGs formed as a result of the enzymatic acidolysis between CB and MSO. The detection of DAGs and PDAGs was limited, mainly due to partial overlap with the free fatty acid band, which hindered their clear visualization and differentiation. In addition, the presence of MAGs suggests that the reaction did not proceed to full completion, reflecting the coexistence of reaction intermediates and residual lipid species. This profile is consistent with an active acyl exchange process under the selected reaction conditions and supports the occurrence of enzymatic remodeling of the lipid matrix.

The color parameters (L*, a*, and b*) of the oils are summarized in Table 7. The MSO exhibited a high luminosity (L* = 89.51 ± 0.02), with a negative a* value (−11.73 ± 0.01) indicating a greenish hue and a positive b* value (39.96 ± 0.02) corresponding to yellow tones. In contrast, the CB showed lower luminosity (L* = 59.21 ± 0.01) and higher positive values for a* (60.31 ± 0.03) and b* (99.16 ± 0.05), reflecting a more intense reddish-yellow coloration. The OBL displayed the highest luminosity (L* = 90.38 ± 0.01), an enhanced green hue (a* = −17.94 ± 0.01), and an intensified yellow component (b* = 42.96 ± 0.03). For edible oils and oil-based systems, consumers typically prefer bright, non-dark, and greenish-yellow to yellow tones rather than very dark or strongly red colors [74,75,76]. From a practical standpoint, the color parameters of the optimized bioactive lipid indicate good consumer acceptance, with moderate lightness and minimal excessive redness or yellowness that could affect visual appeal in food or nutraceutical formulations. Thus, an OBL with moderate lightness and only slight redness/yellowness will visually integrate well into soft gels, beverages, or functional oils without appearing rancid, overly colored, or opaque. These attributes facilitate product standardization across batches, as the OBL maintains chromatic coordinates compatible with clear or lightly colored delivery systems (soft gels, beverages, functional oils) without requiring additional bleaching or masking agents. The slight shift toward neutral tones relative to the starting oils further supports OBL’s versatility for applications where color consistency is critical for commercial viability and regulatory compliance. Consequently, OBL chromatic coordinates that fall within a clear or lightly colored zone reduce or eliminate the need for bleaching/masking, simplify specification setting, and support regulatory and branding requirements for visually consistent functional and nutraceutical products.

In the MSO analyzed, the predominant fatty acids were linoleic (53.55 ± 0.74 g·100 g^−1^ TFAs), oleic (25.62 ± 0.12 g·100 g^−1^ TFAs) and α-linolenic (10.63 ± 0.18 g·100 g^−1^ TFAs) acids. This result is consistent with those reported by Reinoso et al. [33], who also identified these compounds as the major FAs in MSO. Similarly, Sánchez et al. [32] obtained comparable profiles from oils extracted using supercritical CO_2_ and Soxhlet methods. The slight differences observed may be attributed to variations in the raw material or extraction conditions employed.

The CB exhibited EPA (19.01 ± 0.03 g·100 g^−1^ TFAs), linoleic acid (19.48 ± 0.16 g·100 g^−1^ TFAs), and DHA (30.56 ± 0.02 g·100 g^−1^ TFAs) as the predominant fatty acids, with a total EPA + DHA content of 49.57 g·100 g^−1^ TFAs. These results are consistent with those reported by Dovale-Rosabal et al. [7,38] in refined and deodorized salmon oil concentrates produced through urea complexation, where a higher proportion of DHA relative to EPA was also observed. This trend has been attributed to the lower ability of DHA to form inclusion complexes with urea due to its higher degree of unsaturation and longer carbon chain [9,77]. Furthermore, several studies have demonstrated that the urea complexation method is highly effective for the significant increase in the total EPA + DHA content in n-3 LCPUFA concentrates from marine oils, achieving up to fivefold enrichment compared to crude oil [7,8,10,38].

In the OBL analyzed, the predominant fatty acids were EPA (15.97 ± 0.27 g·100 g^−1^ TFAs), DHA (25.31 ± 0.38 g·100 g^−1^ TFAs), and linoleic acid (26.25 ± 0.12 g·100 g^−1^ TFAs), with a total EPA + DHA content of 41.2 g·100 g^−1^ TFAs. The n-6/n-3 ratios were 4.7:1 for MSO, 0.42:1 for CB, and 0.6:1 for the OBL, indicating an adequate balance between both fatty acid families. According to FAO recommendations for daily EPA and DHA intake, only 0.6 g per day of the OBL would be sufficient to achieve the suggested nutritional requirements. The EPA + DHA content achieved in the optimized bioactive lipid in the present study (41.28 g·100 g^−1^ TFAs) compares favorably with values reported in similar enzymatic acidolysis or supercritical CO_2_ systems. Previous work from our research group [43] reported total EPA + DHA levels of approximately 13.1 g·100 g^−1^ TFAs in structured lipids synthesized under optimized SC-CO_2_ conditions using commercial refined salmon oil and an EPA/DHA concentrate, focusing on structured acylglycerols enriched at the *sn*-2 position. In a related study by the same group [38], gallic acid was incorporated into EPA/DHA-enriched lipids in SC-CO_2_, reaching 19.8 g·100 g^−1^ TFAs at the *sn*-2 position. Van Nguyen and Shahidi [78] produced structured lipids via enzymatic acidolysis in *n*-hexane using virgin coconut oil and n-3 LCPUFA, achieving an incorporation rate of 47.04 g·100 g^−1^ TFAs for DHA + EPA under optimal conditions (3.3% *T. lanuginosus* lipase, 42.2 °C, 33.38 h). More recently, another study from our group [26] synthesized antioxidant lipids from rainbow trout belly oil concentrate and cold-pressed maqui seed oil under SC-CO_2_, obtaining EPA + DHA levels of 26.97 g·100 g^−1^ TFAs at 300 bar and 80 °C. Collectively, these comparisons indicate that the present study achieves EPA/DHA enrichments that are consistently within a valuable range or even higher than those reported previously, while simultaneously incorporating multiple tocopherol and tocotrienol homologs from maqui seed oil into the same lipid matrix, representing a key novelty and functional advantage of our system.

Tocopherols and tocotrienols were identified and quantified in the MSO, CB, and OBL, expressed as mg·kg^−1^ oil. In the MSO, the compounds detected in decreasing order of concentration were α-tocopherol (280.95 mg·kg^−1^), β-tocotrienol (89.75 mg·kg^−1^), δ-tocopherol (10.60 mg·kg^−1^), and β-tocopherol (9.17 mg·kg^−1^). These values were higher than those reported by Bastías-Montes et al. [22] for MSO, who found lower concentrations of α-tocopherol (169.33 mg·kg^−1^) and a different distribution of minor tocopherols and tocotrienols, including γ-tocopherol and γ-tocotrienol. In their study, the extraction method significantly influenced the composition, with the Soxhlet technique yielding the lowest tocopherol contents and the chloroform/methanol method showing intermediate values.

Similarly, Fraguela-Meissimilly et al. [31] reported a markedly higher α-tocopherol concentration (444.20 mg·kg^−1^) in maqui biowaste extracts obtained using supercritical fluid extraction, followed by β-tocotrienol (27.43 mg·kg^−1^), γ-tocotrienol (23.02 mg·kg^−1^), γ-tocopherol (18.98 mg·kg^−1^), β-tocopherol (14.79 mg·kg^−1^), and δ-tocopherol (1.14 mg·kg^−1^) compounds. These findings suggest that the enhanced efficiency and mild operational conditions of supercritical CO_2_ extraction favor the recovery of thermosensitive compounds such as tocopherols.

Overall, the results of the present study confirm that α-tocopherol is the predominant compound in MSO, in agreement with previous reports [22,31,32,33]. However, its concentration varies considerably depending on the extraction technique, raw material characteristics, and processing conditions. The absence of γ-tocopherol and γ-tocotrienol, which were detected in other studies [22,31,32,33], may indicate intrinsic compositional differences in the maqui fruit or potential degradation during post-harvest handling and storage.

In the CB, the tocopherol profile showed the following concentrations in increasing order: α-tocopherol (103.85 mg·kg^−1^), δ-tocopherol (22.00 mg·kg^−1^), γ-tocotrienol (20.79 mg·kg^−1^), and γ-tocopherol (7.40 mg·kg^−1^). In comparison, Hamre et al. [78] reported α-tocopherol concentrations ranging from 40 to 45 mg·kg^−1^ and γ-tocopherol levels of approximately 10 mg·kg^−1^ in Atlantic salmon, with minimal variations among different dietary treatments. Similarly, Trullàs et al. [79] found that rainbow trout oils extracted with organic solvents contained higher α-tocopherol concentrations (7.51 mg·kg^−1^) in fish fed with fish oil–based diets, along with lower levels of β- + γ-tocopherols (0.73 mg·kg^−1^). Furthermore, Park et al. [80] demonstrated that supercritical CO_2_ extraction from anchovy oil yielded significantly higher levels of α-tocopherol (29.26 mg·kg^−1^), β- + γ-tocopherol (11.60 mg·kg^−1^), and δ-tocopherol (63.81 mg·kg^−1^), whereas hexane extraction only recovered δ-tocopherol (8.47 mg·kg^−1^). Overall, these findings confirm that tocopherol concentrations in marine-derived oils vary widely depending on species, diet composition, and most notably, the extraction method employed.

In the OBL, the compounds detected were β-tocotrienol (18.48 mg·kg^−1^), γ-tocotrienol (16.36 mg·kg^−1^), γ-tocopherol (6.92 mg·kg^−1^), β-tocopherol (3.54 mg·kg^−1^) and α-tocopherol (0.39 mg·kg^−1^). The markedly lower levels of α-tocopherol relative to β-tocopherol and tocotrienols in the OBL are mechanistically plausible and likely the result of oxidative degradation processes, although this interpretation cannot yet be considered definitive. α-Tocopherol acts as a highly efficient chain-breaking antioxidant and is preferentially consumed during lipid oxidation; consequently, its depletion is widely used as an indicator of oxidative progression in oils and emulsions [81,82]. In stored lipid systems, substantial losses of tocopherols, frequently in the range of 20 to 40 percent, have been reported as they suppress the formation of lipid peroxides and secondary oxidation products such as hexanal [81,82].

The high proportion of tocotrienols is of nutritional and functional importance due to their well-documented antioxidant, anti-inflammatory, and lipid metabolism-modulating properties. Several studies have shown that tocotrienols can inhibit the mevalonate pathway, thereby reducing cholesterol synthesis [83], and may be more effective than α-tocopherol in scavenging free radicals due to their greater interaction with lipid peroxyl radicals [84]. In particular, γ-tocotrienol has shown cardioprotective, anticancer, and lipid-lowering effects [85,86], while β-tocotrienol, although less studied, has demonstrated promising antitumor potential [87].

The lower total concentration of tocopherols and tocotrienols observed in the OBL could be related to the temperature and pressure conditions applied during processing. Shi et al. [88] reported similar trends, observing that higher pressures (200–250 bar) and a progressive increase in temperature in the range of 50–80 °C would lead to a decrease in the concentration of these compounds. Furthermore, the CALB used in the reaction exhibits a greater affinity for long-chain fatty acids, which may influence the final structure of the TAG.

The oxidative quality of the optimal bioactive lipid (OBL) was evaluated through primary and secondary oxidation markers and compared with those of MSO and belly oil concentrate CB. The peroxide value of the OBL (5.61 mEq O_2_·kg^−1^ oil) indicates the formation of primary oxidation products during processing. Although this value is higher than those observed for the raw oils, it remains below the maximum limit established by the Chilean Food Sanitary Regulation for edible oils (<10 mEq O_2_·kg^−1^ oil) [89] and is close to the limit proposed by the Codex Alimentarius for fish oils (≤5 mEq O_2_·kg^−1^ oil) [90]. Similar increases in peroxide values have been reported for structured lipids obtained from polyunsaturated fatty acid–rich substrates, particularly when enzymatic interesterification and thermal handling are involved, even under controlled conditions [91,92].

Secondary oxidation products, assessed by the *p*-anisidine value, remained at moderate levels in the OBL (*p*-AV = 10.08), well below the Codex limit for fish oils (≤20) [90]. When both primary and secondary oxidation products were integrated through the TOTOX index, the OBL reached a value of 21.3, which is within the maximum value established by Codex (≤26) [90]. Although the TOTOX value of the OBL was higher than those of the individual oils used as raw materials, it remained within internationally accepted quality limits, indicating an acceptable overall oxidative status at the time of analysis. TOTOX is widely recognized as a more representative indicator of oxidative history than peroxide value alone, particularly in lipid systems undergoing processing [91,93].

The formation of conjugated dienes and trienes in the OBL was minimal and comparable to that observed in MSO and CB. Low levels of these conjugated compounds are generally associated with early stages of lipid oxidation and are considered desirable in polyunsaturated lipid matrices, as they indicate limited propagation of oxidative reactions [41,91].

The antioxidant-related properties of the oils were evaluated through total phenolic content and H-ORAC assay. MSO exhibited the highest concentration of phenolic compounds, consistent with its botanical origin and cold-press extraction, whereas CB showed negligible levels. The OBL retained an intermediate phenolic content, reflecting the partial incorporation of phenolic compounds from MSO into the final lipid matrix.

In agreement with these results, MSO showed the highest antioxidant capacity as measured by H-ORAC, while the OBL exhibited a lower but measurable value. These findings indicate that antioxidant compounds present in MSO contribute to the radical-scavenging capacity of the OBL. However, it is well established that in vitro antioxidant capacity assays such as ORAC evaluate peroxyl radical–scavenging potential under defined experimental conditions and do not directly predict oxidative stability or shelf life in complex lipid systems [94,95].

Taken together, the combined evaluation of peroxide value, *p*-anisidine value, TOTOX, conjugated dienes and trienes, total phenolic content, and H-ORAC provides a comprehensive assessment of the oxidative status of the synthesized lipid. While the OBL exhibited higher oxidation indices than the raw oils, all measured quality parameters remained within regulatory limits for edible fish oils. These results indicate that the oxidative status of the OBL is acceptable at the time of evaluation, although additional accelerated oxidation assays, such as Rancimat or oxidative stability index (OSI) tests, would be required to further characterize its long-term stability under stressed storage conditions.

The present study did not include a direct stereospecific analysis of fatty acid distribution within the triacylglycerol backbone of the synthesized lipid. Accordingly, the term structured lipid was deliberately avoided, and the product was described as a bioactive lipid obtained through enzymatic modification. While TLC and DSC analyses provided indirect evidence of enzymatic acidolysis through changes in lipid class distribution and thermal behavior, these techniques do not allow determination of the positional incorporation of EPA and DHA. Considering that lipases such as Lipozyme^®^ 435 may exhibit variable regioselectivity and acyl migration depending on reaction conditions, future studies incorporating stereospecific analytical methods would provide deeper insight into fatty acid distribution and potential bioavailability implications.

## 5. Conclusions

The results of this study demonstrated the technical and scientific feasibility of valorizing aquaculture and plant by-products to produce functional lipids enriched with bioactive compounds. Oil was successfully extracted from rainbow trout (*Oncorhynchus mykiss*) belly, and n-3 LCPUFA were concentrated, reaching 19.01 g·100 g^−1^ TFAs of EPA and 30.56 g·100 g^−1^ TFAs of DHA through urea inclusion; such values are higher than those previously reported for similar lipid concentration strategies.

MSO from *Aristotelia chilensis* (Mol.) Stuntz exhibited a high content of α-linolenic (10.63 g·100 g^−1^ TFAs), oleic (25.62 g·100 g^−1^ TFAs), and linoleic (53.55 g·100 g^−1^ TFAs) acids, together with a significant fraction of natural tocopherols. The enzymatic acidolysis catalyzed by CALB under supercritical CO_2_ conditions optimized the process through RSM, identifying optimal conditions at 72.7 °C and 248.9 bar.

The optimal formulation achieved an EPA + DHA content of 41.28 g·100 g^−1^ TFAs and high concentrations of α-, β-tocopherol, and γ-tocotrienol, confirming the successful synthesis of a new BL with potential antioxidant and anti-inflammatory properties.

This study demonstrates the feasibility of producing a novel bioactive lipid through enzymatic acidolysis under supercritical CO_2_, combining EPA/DHA-rich lipids and tocopherol/tocotrienol-containing plant oils derived from agro-industrial by-products. The RSM-optimized conditions (72.7 °C, 248.9 bar, and 2 h) provide a scalable and environmentally friendly process, supported by solvent-free operation, efficient product recovery, and the use of immobilized CALB at a loading compatible with enzyme reuse and continuous or semi-continuous reactor configurations. Although moderate coefficients of determination and significant lack-of-fit in some models reflect the inherent complexity of multi-substrate systems and limit predictive accuracy for individual tocopherol/tocotrienol homologs, the optimized bioactive lipid exhibited a high EPA + DHA content (41.28 g·100 g^−1^ TFAs) and favorable thermal and color properties, supporting its suitability for functional food, nutraceutical, and cosmetic applications. Analytical characterization by TLC, GLC, and HPLC confirmed the successful incorporation of multiple bioactive compounds into triacylglycerol structures, positioning this oil-based lipid as a stable, high-value ingredient from sustainable sources. Future studies should focus on oxidative stability, shelf-life time under accelerated storage, and the in vitro and in vivo bioavailability of the optimized lipid in relevant food matrices. Overall, these results represent a technological innovation with potential applications in the food, nutraceutical, and cosmetic industries, supporting the development of functional ingredients aimed at improving human health.

## Figures and Tables

**Table 1:** Three-level (3^2^) factorial experimental design for the enzymatic acidolysis of cold-pressed maqui seed oil (MSO) and n-3 LCPUFA concentrate from rainbow trout belly oil (CB) under supercritical CO_2_ conditions.

Experiment	Pressure	Temperature
	(bar)	(°C)
1	100	50
2	100	65
3	100	80
4	200	50
5	200	65
6	200	80
7	300	50
8	300	65
9	300	80
10	200	65
11	200	65
12	200	65

**Table 2:** Three-level factorial design (3^2^) using response surface methodology (RSM) for enzymatic acidolysis under supercritical CO_2_ conditions. Effect of temperature and pressure independent variables on EPA, DHA, and EPA + DHA contents.

Independent Variables		Response Variables		
Pressure	Temperature	EPA	DHA	EPA + DHA
(bar)	(°C)	(g·100 g^−1^ TFAs)		
100	50	13.07	18.38	31.45
200	50	17.80	25.47	43.28
300	50	12.76	20.15	32.91
100	65	17.05	22.82	39.87
200	65	15.88	22.43	38.31
300	65	15.40	23.44	38.83
100	80	15.07	16.89	31.96
200	80	15.41	19.75	35.16
300	80	16.43	22.44	38.87
200	65	16.12	22.87	38.99
200	65	15.27	22.26	37.54
200	65	17.54	22.96	40.50

**Table 3:** Three-level factorial design (3^2^) using response surface methodology (RSM) for enzymatic acidolysis under supercritical CO_2_ conditions. Effect of temperature and pressure supercritical CO_2_ independent variables on α-tocopherol, β-tocopherol, β-tocotrienol, γ-tocopherol, γ-tocotrienol, and δ-tocopherol content.

Independent Variables		Response Variables (mg·kg^−1^ Oil)					
Pressure (bar)	Temperature (°C)	α-Tocopherol	β-Tocopherol	β-Tocotrienol	γ-Tocopherol	γ-Tocotrienol	δ-Tocopherol
100	50	0.07	5.04	20.58	8.98	12.53	3.52
200	50	2.34	7.70	23.70	8.50	19.87	8.28
300	50	0.07	26.85	21.48	25.00	8.38	10.40
100	65	1.16	8.46	24.19	6.55	16.34	5.64
200	65	0.82	12.87	42.09	26.01	26.04	3.79
300	65	1.07	19.19	54.84	11.03	42.42	3.31
100	80	0.97	4.34	28.36	16.63	14.76	2.12
200	80	15.50	13.30	39.12	11.97	14.20	1.80
300	80	2.11	5.53	29.23	19.08	9.88	5.31
200	65	0.00	5.19	25.66	12.54	21.06	0.00
200	65	0.00	12.98	29.19	15.16	17.29	0.00
200	65	0.70	6.21	20.11	4.58	14.38	1.25

**Table 4:** Analysis of variance for each response variable in the optimization of the synthesis process of bioactive lipids (BLs) *.

	Effects	Sum of Squares	DF	Mean Square	F-Ratio	*p*-Value
EPA	A: Pressure	0.06000	1	0.06000	0.06	0.8152
	B: Temperature	1.79307	1	1.79307	1.94	0.2577
	AA	5.65813	1	5.65813	6.13	0.0896
	Lack of fit	16.36570	5	3.27315	3.55	0.1632
	Pure error	2.76927	3	0.92309		
	Total (corr.)	26.64620	11			
DHA	A: Pressure	10.50730	1	10.50730	91.79	0.0024
	B: Temperature	4.03440	1	4.03440	35.25	0.0096
	BB	15.64080	1	15.64080	136.64	0.0013
	Lack of fit	32.65880	5	6.53176	57.06	0.0036
	Pure error	0.34340	3	0.11446		
	Total (corr.)	63.18470	11			
EPA + DHA	A: Pressure	8.95482	1	8.95482	5.66	0.0978
	B: Temperature	0.45375	1	0.45375	0.29	0.6295
	AA	16.05570	1	16.05570	10.14	0.0499
	BB	17.80200	1	17.80200	11.25	0.0439
	Lack of fit	83.00710	4	20.75180	13.11	0.0304
	Pure error	4.74890	3	1.58297		
	Total (corr.)	147.93400	11			
α-tocopherol	A: Pressure	0.18515	1	0.18515	0.95	0.4007
	B: Temperature	43.15340	1	43.15340	222.34	0.0007
	AA	36.33670	1	36.33670	187.22	0.0006
	BB	45.15800	1	45.15800	232.67	0.0006
	Lack of fit	98.29390	4	24.57350	126.61	0.0011
	Pure error	0.58225	3	0.19408		
	Total (corr.)	203.51500	11			
β-tocopherol	A: Pressure	189.67500	1	189.67500	10.79	0.0462
	B: Temperature	44.97960	1	44.97960	2.56	0.2079
	Lack of fit	226.05600	6	37.67600	2.14	0.2837
	Pure error	52.72400	3	17.57800		
	Total (corr.)	513.43200	11			
β-tocotrienol	A: Pressure	175.24100	1	175.24100	2.01	0.2511
	B: Temperature	159.68100	1	159.68100	1.83	0.2687
	Lack of Fit	605.54800	6	100.92500	1.16	0.4904
	Pure Error	261.33200	3	87.11080		
	Total (corr.)	1201.80000	11			
γ-tocopherol	A: Pressure	87.86790	1	87.86790	1.12	0.3673
	B: Temperature	4.52228	1	4.52228	0.06	0.8256
	Lack of fit	186.24200	6	31.04030	0.40	0.8458
	Pure error	235.06800	3	78.35590		
	Total (corr.)	513.70000	11			
γ-tocotrienol	A: Pressure	48.49020	1	48.49020	1.91	0.2608
	B: Temperature	0.63810	1	0.63810	0.02	0.8846
	BB	279.59900	1	279.59900	11.02	0.0451
	Lack of fit	498.86000	5	99.77200	3.93	0.1446
	Pure error	76.13360	3	25.37790		
	Total (corr.)	903.71500	11			
δ-tocopherol	A: Pressure	10.01820	1	10.01820	3.14	0.1747
	B: Temperature	28.08220	1	28.08220	8.78	0.0594
	Lack of fit	46.19720	6	10.69950	3.35	0.1743
	Pure error	9.58126	3	3.19375		
	Total (corr.)	111.82500	11			

* The statistically significant effects (*p* < 0.05) are highlighted in red.

**Table 5:** Optimization of enzymatic acidolysis process variables using supercritical CO_2_ by response surface methodology (RSM) to obtain bioactive lipids (BL).

Response Variables	Supercritical CO_2_ Pressure	Supercritical CO_2_ Temperature	Stationary Point	Optimized Values
	**Part a**: Optimization of the process variables			
DHA (g·100 g^−1^ TFAs)	300.00	62.30	Maximum	24.19
EPA + DHA (g·100 g^−1^ TFAs)	224.91	64.20	Maximum	39.98
α-tocopherol (mg·kg^−1^ oil)	202.38	80.00	Maximum	8.65
β-tocopherol (mg·kg^−1^ oil)	300.00	50.00	Maximum	19.00
γ-tocotrienol (mg·kg^−1^ oil)	300.00	64.75	Maximum	25.77
	**Part b**: Multiple response optimization of the response variables and desirability Optimized process variables to obtain BL			
DHA (g·100 g^−1^ TFAs)			Maximum	22.42
EPA + DHA (g·100 g^−1^ TFAs)			Maximum	39.01
α-tocopherol (mg·kg^−1^ oil)	248.92	72.72	Maximum	3.53
β-tocopherol (mg·kg^−1^ oil)			Maximum	11.98
γ-tocotrienol (mg·kg^−1^ oil)			Maximum	21.58
	**Part c**: Experimental validation of the multiple response optimization of the response variables of Part b Optimized process variables by RSM			
DHA (g·100 g^−1^ TFAs)			Maximum	25.31
EPA + DHA (g·100 g^−1^ TFAs)			Maximum	41.28
α-tocopherol (mg·kg^−1^ oil)	248.92	72.72	Maximum	0.39
β-tocopherol (mg·kg^−1^ oil)			Maximum	3.54
γ-tocotrienol (mg·kg^−1^ oil)			Maximum	16.36

**Table 6:** Thermal behavior of cold-pressed maqui seed oil (MSO), n-3 LCPUFA concentrate from trout belly oil (CB) and optimal bioactive lipid (OBL) *.

Oil	T_Onset_ (°C)	T_Peak 1_ (°C)	T_Peak 2_ (°C)	T_Peak 3_ (°C)	T_Endset_ (°C)	ΔH (J·g^−1^)
**MSO**	−75.59 ± 0.08 ^a^	−72.67 ± 0.01 ^a^	−34.74 ± 0.06 ^c^	−23.94 ± 0.06 ^a^	−15.56 ± 0.18 ^b^	37.45 ± 2.34 ^a^
**CB**	−72.09 ± 0.35 ^c^	−66.26 ± 0.48 ^b^	−42.28 ± 0.62 ^a^	-	−39.24 ± 0.72 ^a^	5.73 ± 0.48 ^c^
**OBL**	−73.53 ± 0.23 ^b^	−69.06 ± 0.35 ^b^	−36.57 ± 0.18 ^b^	−5.43 ± 1.58 ^b^	−1.24 ± 0.57 ^c^	10.42 ± 0.08 ^b^

* Values correspond to mean ± standard deviation (*n* = 3). Values followed by different letters in the same column indicate significant differences (*p* < 0.05) according to Tukey HSD.

**Table 7:** Color results L*, a* and b* for cold-pressed maqui seed oil (MSO), n-3 LCPUFA concentrate from trout belly oil (CB) and optimal bioactive lipid (OBL) ^∫^.

Oil	L*	a*	b*
MSO	89.51 ± 0.02 ^a^	−11.73 ± 0.01 ^a^	39.96 ± 0.02 ^a^
CB	59.21 ± 0.01 ^b^	60.31 ± 0.03 ^b^	99.16 ± 0.05 ^b^
O BL	90.38 ± 0.01 ^c^	−17.94 ± 0.01 ^c^	42.96 ± 0.03 ^c^

^∫^ Values correspond to mean ± standard deviation (*n* = 3). Values with different letters in the same column indicate significant differences (*p* < 0.05) according to Tukey HSD.

**Table 8:** Identification and quantification of fatty acids (FA) of Cold-pressed maqui seed oil (MSO), n-3 LCPUFA concentrate from trout belly oil (CB) and Optimal bioactive lipid (OBL) *.

Systematic Name	Abbreviated Name	MSO	CB	OBL
Myristic acid	C14:0	N/I	0.60 ± 0.01 ^a^	0.35 ± 0.07 ^b^
Palmitic acid	C16:0	5.24 ± 0.94 ^a^	1.53 ± 0.02 ^b^	1.43 ± 0.09 ^b^
Palmitelaidic acid	C16:1n-7t	N/I	0.12 ± 0.01 ^a^	0.24 ± 0.23 ^a^
cis-Palmitoleic acid	C16:1n-7	N/I	1.51 ± 0.00 ^a^	0.93 ± 0.11 ^b^
Heptadecanoic acid	C17:0	N/I	0.25 ± 0.00 ^a^	0.24 ± 0.07 ^a^
Heptadecenoic acid	C17:1n-7	N/I	0.12 ± 0.01 ^a^	0.12 ± 0.02 ^a^
Stearic acid	C18:0	3.21 ± 0.45 ^a^	0.92 ± 0.01 ^b^	0.69 ± 0.19 ^b^
Trans-vaccenic acid	C18:1n-7t	N/I	1.63 ± 0.06 ^a^	0.85 ± 0.04 ^b^
Oleic acid	C18:1n-9	25.62 ± 0.12 ^a^	2.89 ± 0.03 ^b^	4.79 ± 0.30 ^c^
cis-Vaccenic acid	C18:1n-7	0.35 ± 0.05 ^a^	0.36 ± 0.04 ^a^	0.13 ± 0.02 ^b^
trans-Linoleic acid	C18:2n-6t	0.10 ± 0.03 ^a^	0.11 ± 0.00 ^a^	0.19 ± 0.20 ^a^
Linoleic acid	C18:2n-6	53.55 ± 0.74 ^a^	19.48 ± 0.16 ^b^	26.25 ± 0.12 ^c^
cis-9-Nonadecenoic acid	C19:1n-10	N/I	0.11 ± 0.00 ^a^	0.04 ± 0.02 ^a^
Arachidic acid	C20:0	0.00 ± 0.01 ^a^	N/I	0.06 ± 0.01 ^b^
γ-Linolenic acid	C18:3n-6	0.28 ± 0.14 ^a^	0.79 ± 0.03 ^b^	0.59 ± 0.03 ^b^
5-Eicosenoic acid	C20:1n-15	0.10 ± 0.07 ^a^	1.09 ± 0.01 ^b^	N/I
8-Eicosenoic acid	C20:1n-12	0.14 ± 0.16 ^a^	3.34 ± 0.06 ^b^	0.97 ± 0.11 ^c^
α-Linolenic acid	C18:3n-3	10.63 ± 0.18 ^a^	3.77 ± 0.05 ^b^	4.39 ± 0.24 ^b^
Eicosadienoic acid	C20:2n-6	N/I	2.31 ± 0.12 ^a^	1.91 ± 0.23 ^a^
Eicosatrienoic acid	C20:3n-3	0.78 ± 0.06 ^a^	N/I	0.30 ± 0.27 ^a^
Docosanoic acid	C22:0	N/I	0.45 ± 0.00 ^a^	0.38 ± 0.06 ^a^
cis-11,14,17-Eicosatrienoic acid	C20:3n-3	N/I	1.30 ± 0.07 ^a^	1.28 ± 0.27 ^a^
Arachidonic acid	C20:4n-6	N/I	0.19 ± 0.01 ^a^	0.26 ± 0.24 ^a^
Docosadienoic acid	C22:2n-6	N/I	2.30 ± 0.07 ^a^	1.97 ± 0.12 ^a^
Eicosapentaenoic acid (EPA)	C20:5n-3 (EPA)	N/I	19.01 ± 0.03 ^a^	15.97 ± 0.27 ^b^
Docosatrienoic acid	C22:3n-3	N/I	1.51 ± 0.01 ^a^	1.39 ± 0.02 ^b^
Docosatetraenoic acid	C22:4n-3	N/I	0.51 ± 0.00 ^a^	0.54 ± 0.09 ^a^
Docosapentaenoic acid (DPA)	C22:5n-3 (DPA)	N/I	3.23 ± 0.00 ^a^	2.34 ± 0.03 ^b^
Docosahexaenoic acid (DHA)	C22:6n-3 (DHA)	N/I	30.56 ± 0.02 ^a^	25.31 ± 0.38 ^b^
Total saturated fatty acids		8.45	3.76	3.15
Total monounsaturated fatty acids		26.21	11.18	8.08
Total polyunsaturated fatty acids		65.34	85.86	82.70
Total n-3 polyunsaturated fatty acids		11.41	59.89	51.53
EPA + DHA		N/I	49.57	41.28

* Values correspond to mean ± standard deviation (*n* = 3). Values with different letters indicate significant differences (*p* < 0.05) according to Tukey HSD. N/I = Not Identified.

**Table 9:** Identification and quantification of tocopherols and tocotrienols in cold-pressed maqui seed oil (MSO), n-3 LCPUFA concentrate from trout belly oil (CB) and optimal bioactive lipid (OBL) *.

Oil	α-Tocopherol	β-Tocopherol	β-Tocotrienol	γ-Tocopherol	γ-Tocotrienol	δ-Tocopherol
**MSO**	280.95 ± 0.12 ^a^	9.17 ± 0.08 ^a^	89.75 ± 0.13 ^a^	N/I	N/I	10.60 ± 0.20 ^a^
**CB**	103.85 ± 1.27 ^b^	N/I	N/I	7.40 ± 0.29 ^a^	20.79 ± 0.05 ^a^	22.00 ± 0.01 ^b^
**OBL**	0.39 ± 0.08 ^c^	3.54 ± 0.30 ^b^	18.48 ± 0.37 ^b^	6.92 ± 0.09 ^a^	16.36 ± 3.12 ^a^	N/I

* Values correspond to mean ± standard deviation (*n* = 2). Values with different letters indicate significant differences (*p* < 0.05) according to Tukey HSD. N/I = Not Identified.

**Table 10:** Chemical analysis results of cold-pressed maqui seed oil (MSO), belly oil from rainbow trout (*Oncorhynchus mykiss*) (RTBO), and the optimal bioactive lipid (OBL).

Analysis	MSO *	RTBO *	OBL
Peroxide value (mEq O_2_·kg^−1^ oil)	0.23 ± 0.06 ^a^	0.31 ± 0.03 ^a^	5.61 ± 0.25 ^b^
Free fatty acids (FFA; g oleic acid·100 g^−1^ oil)	0.22 ± 0.04 ^a^	0.16 ± 0.02 ^b^	N.D.
*p*-Anisidine value (*p*-AV, units)	9.12 ± 0.00 ^a^	6.86 ± 0.03 ^b^	10.08 ± 0.12 ^c^
Conjugated dienes	0.05 ± 0.00 ^a^	0.06 ± 0.02 ^a^	0.01 ± 0.03 ^a^
Conjugated trienes	0.02 ± 0.00 ^a^	0.02 ± 0.00 ^a^	0.01 ± 0.03 ^a^
TOTOX value	9.58 ± 0.12 ^a^	7.49 ± 0.06 ^b^	21.3 ± 0.30 ^c^

Values are expressed as mean ± standard deviation (*n* = 3). Values with different letters indicate significant differences (*p* < 0.05) according to Tukey HSD. * Reinoso et al. [33].

**Table 11:** Total phenolic content of cold-pressed maqui seed oil (MSO), belly oil from rainbow trout (*Oncorhynchus mykiss*) concentrate (CB), and the optimal bioactive lipid (OBL).

Sample	Total Phenolic Content (mg GAE·g^−1^ Oil)
MSO *	2.871 ± 0.01 ^a^
CB	0.028 ± 0.43 ^c^
OBL	0.743 ± 0.22 ^b^

Values are expressed as mean ± standard deviation (*n* = 3). Values with different letters indicate significant differences (*p* < 0.05) according to Tukey HSD. * Reinoso et al., 2024 [33].

**Table 12:** H-ORAC-FL value of cold-pressed maqui seed oil (MSO), belly oil from rainbow trout (*Oncorhynchus mykiss*) concentrate (CB), and the optimal bioactive lipid (OBL).

Sample	Antioxidant Capacity (µmol Trolox Equivalents (TE)·g^−1^ Oil)
MSO *	4.85 ± 0.00 ^a^
CB	N/D
OBL	1.08 ± 0.00 ^b^

Values are expressed as mean ± standard deviation (*n* = 3). Values with different letters indicate significant differences (*p* < 0.05) according to Student’s *t*-test. * Sanchez et al. [32].

## Data Availability

All the data are contained within the manuscript.

## Abbreviations

The following abbreviations are used in this manuscript:

n-3 LCPUFA	Long chain polyunsaturated fatty acids omega-3
ALA	Alpha-linolenic acid
EPA	Eicosapentaenoic acid
DHA	Docosahexaenoic acid
SFAs	Saturated fatty acids
MUFAs	Monounsaturated fatty acids
TAGs	Triacylglycerols
FFAs	Free fatty acids
BLs	Bioactive lipids
CALB	*Candida antarctica* lipase B
GRAS	Generally Recognized As Safe
MSO	Cold-pressed maqui seed oil
GLC	Gas–liquid chromatography
RSM	Response Surface Methodology
CB	n-3 LCPUFA concentrate from trout belly oil
TFAs	Total fatty acids
HPLC	High-performance liquid chromatography
OBL	Optimal bioactive lipid
ANOVA	Analysis of variance
DSC	Differential scanning calorimetry
CIE	International Commission on Illumination
FAMEs	Fatty acid methyl esters
IUPAC	International Union of Pure and Applied Chemistry
AOCS	American Oil Chemistry Society
TLC	Thin-layer chromatography
MAGs	Monoacylglycerols
DAGs	Diacylglycerols
SFC	Solid fat content
PDAG	Phenolic diacylglycerols
